# The Potential of Electrospun Membranes in the Treatment of Textile Wastewater: A Review

**DOI:** 10.3390/polym16060801

**Published:** 2024-03-13

**Authors:** Joana M. Rocha, Rui P. C. L. Sousa, Raul Fangueiro, Diana P. Ferreira

**Affiliations:** Centre for Textile Science and Technology (2C2T), University of Minho, 4800 Guimarães, Portugal; b13622@2c2t.uminho.pt (J.M.R.); rp.cls@hotmail.com (R.P.C.L.S.); rfangueiro@det.uminho.pt (R.F.)

**Keywords:** adsorbents, electrospinning, fibrous materials, industries, wastewater treatment

## Abstract

Water security and industrial wastewater treatment are significant global concerns. One of the main issues with environmental contamination has been the discharge of dye wastewater from the textile and dye industries, contributing to an ever-growing problem with water pollution, poisoning water supplies, and harming the ecosystem. The traditional approach to wastewater treatment has been found to be inefficient, and biosorption techniques and mechanisms have been proven to be a successful replacement for conventional methods. Recent developments have led to the recognition of fibrous materials as an environmentally friendly option with broad application in several industries, including wastewater treatment. This review explores the potential of fibrous materials produced by the electrospinning technique as adsorbents for wastewater treatment, while at the same time, for the removal of adsorbates such as oil, dyes, heavy metals, and other substances, as reported in the literature. Textile wastewater filtering structures, produced by electrospinning, are summarized and the use of synthetic and natural polymers for this purpose is discussed. The limitations of electrospun textile wastewater filtering structures are also mentioned. Electrospun nanofibrous membranes appear to be a very promising route to filter textile wastewater and therefore contribute to water reuse and to reducing the contamination of water courses.

## 1. Introduction

Water pollution is one of the many environmental issues that have recently surfaced as a result of overcrowding, urbanization, an increase in various industrial and human activities, the growth of landfills, mining activities, and urban wastewater [1,2]. Huge quantities of different toxic pollutants are discharged into water resources on a daily basis, causing increasingly serious water pollution problems, contaminating water bodies, and damaging the environment [2,3]. Therefore, water supplies are contaminated with a variety of pollutants, including oil, microbes, viruses, pharmaceutical waste, organic and inorganic pollutants, and nanoparticles [4]. Dyes and heavy metal ions are considered the main water pollutants [4]. Water contamination is one of the most important worldwide issues at the moment because there are not enough water sources. By 2025, around 3.5 billion people worldwide will face a shortage of fresh water [5]. As a result, researchers are working to create sustainable, affordable, and alternative solutions for treating or recycling wastewater [6].

The particular case of the textile industry is alarming. This industry is one of the major contributors to effluent wastewater due to the huge consumption of water in different parts of the process. It is estimated that the textile industry consumes about 200 L of water per kilogram of fabric processed, with these processes generating around 17 to 20% of total industrial wastewater [7,8]. In the wet fabric processing industry, processing operations include steps such as scouring, bleaching, dying, printing, and finishing stages, which generate different pollutants in the wastewater. Effluent wastewater may contain chemicals like alkalis, reactive dyes and other organic compounds, hydrogen peroxide, surfactants, or metal ions [9]. It has been estimated that each year, approximately 100 tons of dyes are released into drinking water [10]. Therefore, a variety of treatment methods, including physical (adsorption and filtration), oxidation (ozone, H_2_O_2_, or Fenton’s process), biological (fungi, algae, or bacteria), and hybrid processes, have been developed to effectively treat textile wastewater to avoid the discharge of the mentioned pollutants [11]. However, many innovative technologies are emerging to optimize and improve the current methods in terms of recycling, reducing costs, and increasing sustainability.

With a focus on producing nanomaterials with particular characteristics for use in a range of water purification applications, nanotechnologies and nanomaterial sciences have received a lot of attention recently. Of all of the nanostructured materials currently available, nanofibers have proven to have unique qualities and characteristics that allow them to overcome the constraints of traditional fibrous structures [12]. One of the most significant methods for producing these nanomaterials is electrospinning. It is a sophisticated method for creating continuous fibers that range from micro- to nanoscale in size. Furthermore, it is an adaptable method that works with a variety of spinnable materials, is accurate and controllable, and has an excellent cost-effectiveness ratio [13]. Generally, an electric field strong enough to overcome the surface tension of the polymer material is created by applying an electrostatic force over a predetermined distance between a collector and a syringe filled with a polymer solution. As a result, the polymer solution is transformed into a fiber structure that is deposited on the collector, creating a non-woven mat with good mechanical qualities, a high surface area, a low weight connected porous structure, and high porosity. In order to tailor nanofiber mats for particular applications [14], functional additives such as surface coating, interfacial polymerization (IP), or active species can be added to the electrospinning solution [15,16,17]. These modification techniques provide electrospun nanofibers with strong advantages for different applications, such as water purification. 

The utilization of electrospinning techniques to create nanofibrous structures has demonstrated significant promise in wastewater treatment because of its advantageous properties for the elimination of both organic and inorganic contaminants from aqueous systems [2,5,10,18,19]. The abovementioned superior characteristics of these structures and the tunability of the electrospinning process turn electrospun nanostructures into a promising route for wastewater treatment. Additionally, through employing such cutting-edge methods and scalable production processes, society can become less dependent on conventional purifying technologies and cut down on the resources and energy that these technologies consume [18].

Despite some reports on water purification approaches with various materials and techniques, the application of different types of nanofibers obtained by the electrospinning technique in water purification systems is quite limited. In this work, the potential of filtering electrospun structures for textile wastewater treatment is discussed. An overview of the electrospinning process and its processing parameters is given. The reports on filtering structures obtained by electrospinning are reviewed and the limitations of this field are summarized.

## 2. Textile Wastewater: Composition and Purification Requirements

**Figure 1:**
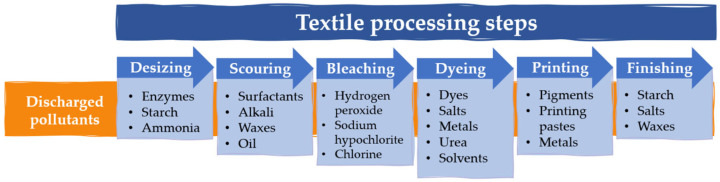
Textile processing steps and pollutants the may be discharged in each step.

The composition of textile wastewater is complex and diverse, with substantial limitations to successful pollutant treatment and removal. Textile wastewater comprises several types of contaminants, with textile dyes being particularly problematic given their xenobiotic properties [20]. Furthermore, organic chemicals including aldehydes, alcohols, ketones, and surfactants add to the complexity of textile effluent. Wastewater is generated during textile processing steps, such as dyeing, printing, and finishing (Figure 1) [21]. The discharge of wastewater from the textile industry without adequate treatment can result in the discharge of pollutants that include carcinogens, mutagens, or heavy metals, endangering human health and aquatic environments [22]. 

Textile wastewater’s physicochemical features, such as high amounts of suspended substances, a high chemical oxygen demand, acidity, heat, and color, emphasize the wide range of contaminants found in its composition [21]. While oxidation and biological methods, such as ozonation, Fenton’s process, or bacterial treatment, have been widely explored for the removal of textile wastewater constituents [23], physical methods such as the use of porous membranes with high selectivity and efficiency may be the solution for the adsorption/filtration of dyes, other organic compounds, and heavy metals.

**Figure 2:**
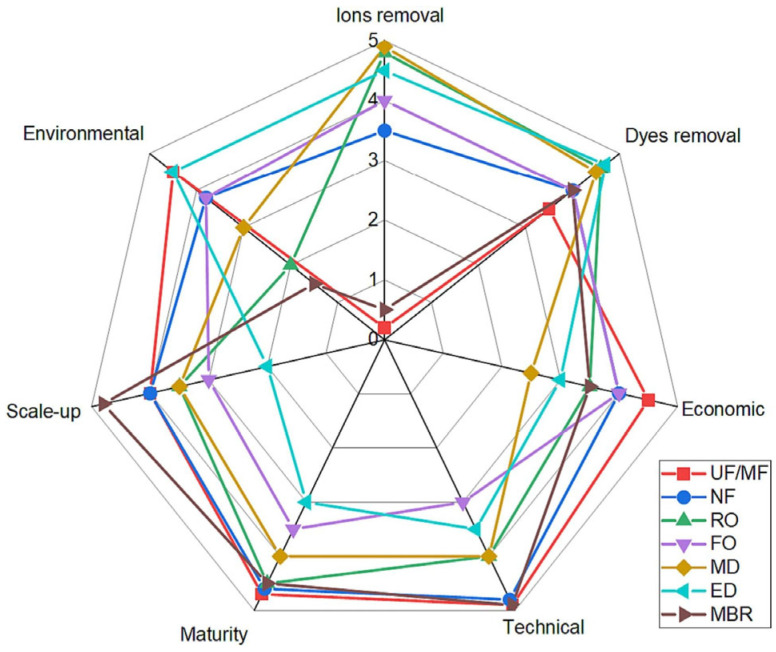
Comparative analysis of the membrane technologies in textile wastewater treatment, with an evaluation of the following parameters: ion removal efficiency, dye removal efficiency, economic analysis, technical feasibility, maturity, scalability, and environmental analysis. The scale indicates the rating from lowest to highest, with a minimum of 1 and a maximum of 5 points. Reprinted with permission from reference [25].

Membrane separation processes include microfiltration, ultrafiltration, nanofiltration, and reverse osmosis [24]. Other membrane technologies have been recently used for wastewater treatment, such as forward osmosis, membrane distillation, electrodialysis, or membrane bioreactors. A comparative analysis of the different membrane technologies was carried out by Ma et al. with the help of the software Circos (version 0.69–6) (Figure 2) [25]. Fibrous membranes exhibit tunable pore sizes that can act in some of the mentioned processes, as the effective separation of components smaller than the pore size is essential for efficient filtration [26]. Indeed, membrane separation can not only efficiently remove the mentioned contaminants, but also adsorb and recycle dyes and other organic chemicals for reuse in the textile processing.

The effectiveness of membranes for wastewater adsorption or filtration relies on specific characteristics. Membranes’ tunable nature, porosity, surface area-to-volume ratio, morphology, tensile strength and elongation, and wettability play crucial roles in determining their suitability for filtration applications. Electrospun membranes show structural and mechanical features which render them potentially advantageous for application in the filtration of textile wastewater, such as their high porosity, large surface area-to-volume ratio, and mechanical stability.

## 3. Electrospinning—Process Technology and Operation

Electrospinning is a simple, fast, and versatile technique that uses electrostatic forces to produce fibers with controllable diameters from polymer solutions [27]. Fibers produced by electrospinning are characterized by their small diameters, which can vary from nanometers to micrometers, their high surface-to-volume ratio, high porosity, easy surface functionalization, and high flexibility to tune the shape and size [28,29]. Furthermore, this technique allows for the use of different polymeric solutions, made up of natural and/or synthetic polymers, which can also incorporate reinforcing agents and bioactive agents, allowing for the production of structures with advanced properties. These characteristics mean that nanofibers produced by electrospinning are of potential interest for applications in various areas, namely in the textile industry for military protective clothing, in environmental engineering for environmental monitoring, in tissue engineering for the development of structures or supports, and in biomedical applications, for dressings with the release of active ingredients [30,31]. Control of the morphology and final dimensions of the produced fibers depends on the different processing parameters, which include the parameters of the polymer solution, the process/equipment, and also the environmental parameters [32].

**Figure 3:**
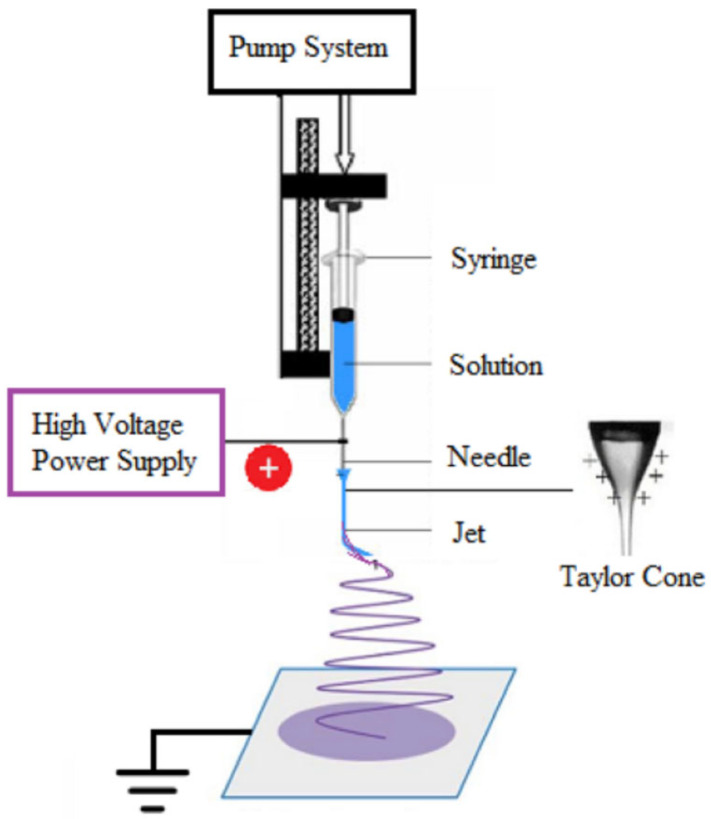
Schematic representation of electrospinning equipment. Reproduced with permission from reference [35].

The fundamental arrangement of electrospinning apparatus is shown schematically in Figure 3. It has three main components: a grounded metal collector, a capillary tube with a smalldiameter metal needle coupled to a high voltage source, and a syringe containing the polymer solution [33,34]. 

In the electrospinning process, the polymer solution is ejected from the needle into the metal collector by applying high voltage between the needle and the collector. In detail, the production of fibers by using electrospinning begins with the transfer of the polymer solution, which is stored in the syringe, through the capillary tube to the needle. Subsequently, the applied electric field induces charges in the polymer solution, which are evenly distributed over the entire surface of the drop at the tip of the needle. As the intensity of the electric field increases, the polymer droplet elongates, acquiring a conical shape commonly referred to as the Taylor cone. When the applied potential reaches the critical value necessary to overcome the surface tension of the polymer droplet, a jet of electrically charged fluid is ejected from the tip of the Taylor cone. As the polymer jet travels from the needle to the collector, the solvent gradually evaporates, and the polymer fibers are deposited on the collector [36,37].

### 3.1. Processing Parameters

Several processing parameters have a major impact on the electrospinning process and can change the morphology, diameter, and shape of the fibers that are produced. There are three main groups for this set of parameters: environmental, process, and solution parameters [38].

#### 3.1.1. Solution Parameters

Solution parameters such as polymer molecular weight, concentration, viscosity, and electrical conductivity directly influence the morphology, geometry, and size of the fibers produced by electrospinning. These parameters are related to the physicochemical properties of the polymers and solvents, as well as the interactions between them [39,40].

- Concentration and viscosity

The concentration of the polymer in the solution and the viscosity of the solution play an important role in the formation of fibers during the electrospinning process. Low concentrations usually cause the polymer’s molecular chains to break down before reaching the collector, leading to the appearance of defects, namely droplets, along with the deposited fibers. On the other hand, the use of high concentrations, combined with solutions with greater viscosity, promotes the production of fibers with fewer defects, as there is greater conjugation of the polymer molecular chains in the solution, favoring the production of a continuous jet. In addition, solutions with a higher viscosity are associated with the production of more uniform fibers with larger diameters [29,34].

**Figure 4:**
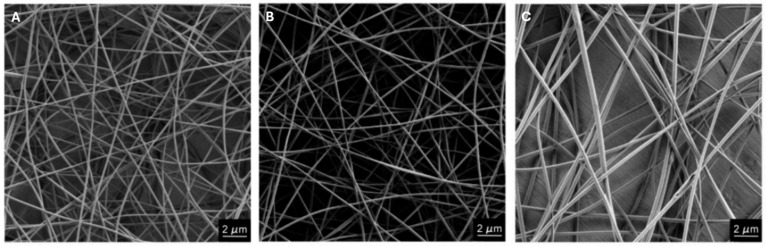
FESEM images of PEO and alginate nanofibers with a total polymer percentage of (**A**) 2.5% (m/m); (**B**) 3.5% (m/m); and (**C**) 4.5% (m/m), using a PEO with a molecular weight of 2 MDa and a proportion of 15% (m/m). Reprinted with permission from reference [41].

Mirtic et al. evaluated the influence of the total concentration of the polymer (PEO and alginate) in the solution on the morphology of the nanofibers developed by electrospinning. To this end, they tested three percentages, 2.5%, 3.5%, and 4.5%, and found that with higher concentrations, nanofibers with proportionally larger diameters were obtained and defects were eliminated (Figure 4) [41]. Similar results were obtained by Dhandayuthapani et al., who evaluated the effect of the concentration of CS and gel on the production of CS nanofibers and gel nanofibers. The authors observed that the use of low concentrations of both polymers, associated with low viscosity, resulted in the production of non-uniform nanofibers with numerous defects. As the concentration of the polymers in the solution increased, it was possible to obtain uniform nanofibers without defects [42]. Finally, Nezarati et al. evaluated the effect of the concentration and viscosity of the polycarbonate urethane (PCU) polymer solution on the morphology of the fibers. Firstly, they found that increasing the concentration of PCU in the solution led to an increase in its viscosity. They also showed that low viscosity values (7.2 ± 1.7 Pa.s), resulting from lower concentration solutions, led to fibers with defects in their structure. Intermediate viscosity values (10.1 ± 0.5 Pa.s) allowed for the development of defect-free fibers with homogeneous diameters. Finally, for high viscosity values (22.5 ± 1.4 Pa.s), there was a substantial increase in the average diameter of the fibers produced, increasing from 1.2 μm to 3.5 μm [43].

Thus, considering the studies presented above, it can be concluded that low polymer concentrations, and consequently reduced viscosity, promote the appearance of defects in the nanofibers produced, while increasing the concentration favors the development of more uniform nanofibers with fewer defects and a larger diameter. However, excessively high concentrations may prevent the solution from flowing through the needle tip and may inhibit fiber formation due to an excessive increase in the solution’s viscosity. It is therefore necessary to find the ideal polymer concentration at which the solution reaches viscosity values suitable for fiber formation in the electrospinning process [29,44].

- Molecular weight

The morphology of fibers created by electrospinning is also significantly influenced by the molecular weight of the polymer. The conjugation of the polymer chains reflects the molecular weight, which in turn affects the solution’s viscosity [45]. Sohi et al. studied the effect of the molecular weight of chitosan (CS) on the production of CS/PEO fibers, where three molecular weights were evaluated: high ((3.10–3.75) × 10^5^ g/mol), medium ((1.90–3.1) × 10^5^ g/mol), and low molecular weight ((0.5–1.9) × 10^5^ g/mol). Initially, using CS solutions of different concentrations and different molecular weights, the authors found that when using 1.5% (*w*/*v*) and 2% (*w*/*v*) CS, it was not possible to produce fibers using medium and low molecular weight CS. In addition, with high molecular weight CS, it was not possible to produce fibers with polymer concentrations in the solution lower than 1.5% (*m*/*v*). Therefore, PEO was incorporated in different proportions in order to improve the properties of the solution for use in electrospinning. From this point, the authors only evaluated the effect of low and medium molecular weight CS. Thus, for a polymer percentage of 2% (*m*/*v*) and a CS/PEO ratio of 1:3, the authors obtained defect-free fibers using low molecular weight CS. With medium molecular weight CS, some defects were observed along with the fibers produced [46]. Similarly, Roldán et al. evaluated the morphology of PCL fibers obtained using different polymer molecular weights, 14,000, 45,000, and 80,000 g/mol. For a polymer percentage of 15% in a solution of acetone and acetic acid (3:7), the authors found that with low molecular weight PCL, no fibers were formed by the electrospinning process. On the other hand, with PCL of 45,000 g/mol, fibers were formed along with the appearance of some droplets. Increasing the molecular weight to 80,000 g/mol allowed for the development of uniform fibers without defects in their structure, although with larger diameters [47]. The effect of the polymer polyvinyl alcohol (PVA) with different molecular weights was also evaluated by Akduman et al., where identical results were observed. Three molecular weights were used, 89,000–98,000 g/mol, 125,000 g/mol, and 146,000–186,000 g/mol. The most uniform fibers were obtained when high molecular weight PVA was used, where no formation of defects in the fiber was observed, unlike for low molecular weight PVA [48].

Generally, high molecular weight polymers are used in the electrospinning process, as they provide the desired viscosity for fiber formation. However, the use of low molecular weight polymers may be sufficient to provide adequate viscosities and thus guarantee the formation of a stable and uniform jet during the electrospinning process. Furthermore, the conjugation of two or more polymers can also facilitate the electrospinning process and thus enable the use of polymers with lower molecular weights [49].

- Conductivity

**Figure 5:**
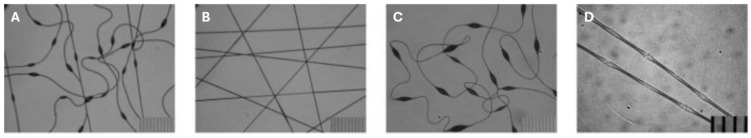
FESEM images of PHBV fibers with different solvents, (**A**) chloroform; (**B**) chloroform/alcohol (3:1); (**C**) chloroform/carbon tetrachloride (3:1); (**D**) chloroform/dimethylformamide (3:1), using a voltage of 22 kV and a feed rate of 4 mL/h. Reproduced with permission from reference [51].

The conductivity of the solution is mainly determined by the type of polymer, solvent, and the availability of ionizable salts. A solution with high electrical conductivity will have a greater capacity to transport charges than one with low conductivity. Thus, raising the solution’s conductivity to a crucial value will raise the charge on the polymer droplet’s surface and promote the Taylor cone’s development. Several authors have verified the existence of an inverse proportional relationship between the electrical conductivity and the diameter of the fibers produced by electrospinning. Thus, higher conductivity values result in a smaller fiber diameter, while low conductivity leads to the formation of fibers with larger diameters due to insufficient stretching of the polymer jet [29,50]. A study carried out by Zuo et al. using the polymer poly(3-hydroxybutyrate-co-3-hydroxyvalerate) (PHBV) showed that the conductivity of the solution also has a major influence on the formation of defects in nanofibers produced by electrospinning. In addition, the authors found that the conductivity of the solution varies with the type of solvent used. In fact, the addition of alcohol to the PHBV solution in chloroform promoted an increase in the solution’s conductivity from 0.3190 to 13.50 μS/cm, resulting in the formation of more uniform fibers with no defects in their structure. On the other hand, the incorporation of carbon tetrachloride into the PHBV/chloroform solution decreased the solution’s conductivity values (0.01700 μS/cm) and consequently favored the appearance of various defects along the deposited fibers. Finally, the addition of dimethylformamide to the PHBV/chloroform solution significantly increased the conductivity (22.50 μS/cm), and smooth fibers with fewer defects were produced (Figure 5) [51].

Amariei et al. also showed that the electrical conductivity of the solutions is a determining factor in the diameter of the fibers produced by electrospinning. In this study, 10% PVA was dissolved in different solvents, water, ethanol (20% and 50%), and acetic acid (20% and 50%). The highest electrical conductivity value, 1.39 mS/cm, was obtained with 20% acetic acid, resulting in fibers with diameters of 82 ± 16 nm. The conductivity values decreased with the use of 50% acetic acid, water, and 20% ethanol, respectively. Finally, the solution with the lowest conductivity value (0.54 mS/cm), obtained with 50% ethanol, produced less uniform fibers with larger diameters (955 ± 123 nm) [52]. It can be seen that increasing the electrical conductivity of the solution leads to greater uniformity and a decrease in the diameter of the fibers, while at the same time reducing the number of associated defects [53].

#### 3.1.2. Process Parameters

Included in the process parameters are all of the variables related to the electrospinning apparatus, such as the voltage applied, the distance between the needle and the collector, the feed rate, the needle diameter, and the kind of collector utilized. The user chooses these parameters based on the type of solution to be used, and each one directly affects the diameter and shape of the fibers that are developed [29,54].

- Applied voltage

The applied tension is a parameter that can be manipulated in order to control the quality and diameter of the fibers produced during the electrospinning process. In this process, sufficiently strong tension is required to overcome the surface tension of the polymer droplet and thus cause a jet to form through the Taylor cone [38]. In this way, very low-tension values may not be sufficient for the correct formation of the Taylor cone, leading to the formation of droplets hanging from the tip of the needle [45]. 

Haghju et al. evaluated the effect of different voltage values, 20 and 30 kV, on the production of CS/PVA fibers by electrospinning. For a constant polymer concentration in the solution, applying a voltage of 20 kV produced fibers with diameters of 522.55 nm and 365.64 nm for feed flow rates of 0.1 and 0.5 mL/h, respectively. As the voltage increased to 30 kV, there was a decrease in fiber diameter to 290.38–405.62 nm and 162.19–318.97 nm, depending on the feed rate. On the other hand, lower values of applied voltage resulted in more homogeneous fibers with fewer defects in their structure [55]. The influence of the applied tension on the morphology and diameter of Eudragite^®^ L100 fibers produced by electrospinning was also evaluated by Reda et al. Four different voltages were tested, 10, 15, 20, and 25 kV. At a voltage of 10 kV, fibers with average diameters of 123.91 ± 48.35 nm were produced. As the voltage value increased to 15, 20, and 25 kV, a progressive increase in fiber diameter was observed to 125.58 ± 33.19, 182.75 ± 62.60, and 186.43 ± 51.01 nm, respectively [56]. 

The studies described above show that the relationship between the tension applied and the diameter of the fibers produced by electrospinning is ambiguous. In this sense, the tension applied must be adjusted to appropriate values according to the composition of the solution under study in order to guarantee a continuous jet.

- Distance between the needle and collector

The distance between the needle and the collector significantly influences the shape and size of the fibers created by electrospinning. This parameter has a direct influence on the deposition time, the solvent evaporation rate, and the stability of the jet, so a minimum distance must be found that allows the fibers to dry properly before they reach the collector. In addition, the distance will always be related to the concentration of the polymer solution and the applied voltage [57]. 

Fallah et al. studied the influence of the distance between the needle and the collector in PCL and gelatin fibers [58]. Three different distances were tested (10, 18, and 21 cm), with the feed rate and applied tension remaining constant. At a distance of 10 cm, fibers with diameters of around 130 nm were obtained. As the distance increased to 18 cm, smaller diameters were obtained (95 nm). However, when the distance was increased to 21 cm, the diameter increased again. This result is due to the fact that when the distance increases from 10 cm to 18 cm, the solvent has more time to evaporate before reaching the collector and, consequently, the final diameter of the fibers is reduced. However, when the distance increases to 21 cm, there is a reduction in the electrostatic force on the ejected jet, preventing the jet and the resulting nanofibers from being sufficiently elongated, resulting in larger diameters. Similar results were obtained by Doshi et al. in their study of another polymeric system [59]. 

Thus, using the results presented by the previous studies, it is possible to conclude that there is a minimum and maximum limit for the value of the distance between the needle and the collector in order to reduce the diameter of the fiber to a minimum value.

- Flow rate

The flow rate of the polymer solution is an important parameter in the electrospinning process, as it influences the speed of the jet and the transfer rate of the polymer solution. To achieve proper evaporation of the solvent and obtain smooth (defect-free) nanofibers, lower feed rates are generally preferable. On the other hand, excessively low flow rates can lead to the unavailability of the solution at the needle tip. However, because the solvent does not completely evaporate before reaching the collector, using extremely high flow rates might lead to the generation of fibers with greater diameters and structural flaws [31,60]. 

Kyselica et al. studied the influence of applying different flow rates to a PEO solution. In this study, the authors found that low flow rates, but that were sufficient to supply the polymer solution to form the Taylor cone, were the ones that allowed for the formation of nanofibers with the fewest defects. On the other hand, feed rates that were too low led to interruptions in the jet due to the unavailability of the solution at the tip of the needle, while high flow rates led to dripping and the production of thicker fibers [32].

**Figure 6:**
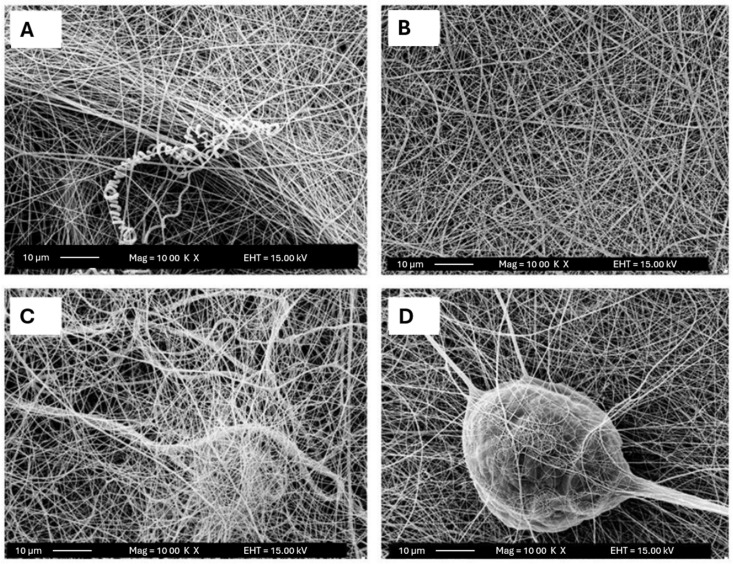
FESEM images of Nylon 6 fibers produced by electrospinning, applying different feed flow rates: (**A**) 0.1 mL/h; (**B**) 0.5 mL/h; (**C**) 1 mL/h; and (**D**) 1.5 mL/h. Adapted with permission from reference [61].

Similar outcomes were obtained by Zargham et al. in their study which varied the feed rate between 0.1 and 1.5 mL/h in Nylon 6 solutions. When a flow rate of 0.5 mL/h was applied at a voltage of 29 kV, with a polymer content of 20% in the solution and a distance of 15 cm between the needle and the collector, uniform, flawless fibers with diameters of about 237 nm were obtained. Values below (0.1 mL/h) and above (1 mL/h) this flow rate favored the development of fibers with more heterogeneous diameters and more defects in their structure and, in the case of greater tension, higher average diameters. In addition, the application of an excessively high flow rate (1.5 mL/h) led to the formation of an unstable jet, with a tendency to form fibers with larger and more heterogeneous diameters, while at the same time resulting in a lower density of deposited fiber area (Figure 6) [61].

It can therefore be concluded that the value of the flow rate must be defined in such a way as to ensure the necessary time for the solvent to evaporate properly, while at the same time being sufficient to guarantee the correct availability of the solution at the tip of the needle [45].

- Needle diameter

Although it is not a parameter widely evaluated by researchers, the diameter of the needle also has a significant influence on the morphology of the fibers produced by electrospinning [62].

**Figure 7:**
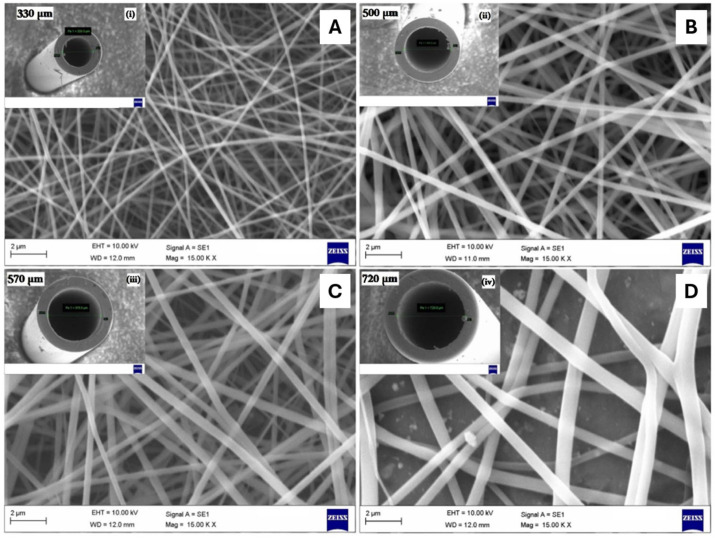
FESEM images of PVP and TiO_2_ fibers using needles with different diameters: (**A**) 330 μm; (**B**) 500 μm; (**C**) 570 μm; and (**D**) 720 μm. Inset: diameter measurements: (**i**) 330 μm; (**ii**) 500 μm; (**iii**) 570 μm; and (**iv**) 720 μm. Adapted with permission from reference [63].

Pisani et al. evaluated the effect of using needles with different diameters, 18 G (1200 μm) and 27 G (400 μm), on PCL/Polylactic Acid (PLA) fibers. In this study, for a total polymer percentage of 10%, the use of the smaller diameter needle led to the development of fibers with numerous defects in their structure. In contrast, the 27 G needle led to the development of defect-free fibers with good morphology and diameters of around 200 nm. For the higher percentages of polymer used (15, 20, and 25%), fibers with good morphology were developed for the different needle diameters used. However, the smaller diameter needle showed fibers with smaller diameters and greater membrane porosity when compared to the larger diameter needle [38]. Similarly, Kuchi et al. tested needles with different diameters, 330, 500, 570, and 720 μm, for the production of polyvinylpyrrolidone (PVP) fibers with titanium dioxide (TiO_2_). Fibers with an average diameter of 150 ± 20 nm were created using the needle with the smallest diameter. The average diameter of the fibers increased to 200 ± 20, 250 ± 20, and 350 ± 20 nm, respectively, when the needle’s diameter was increased to 500, 570, and 720 μm (Figure 7) [63].

Thus, smaller needle diameters not only favor the development of fibers with smaller diameters and without defects, but also increase the porosity of the membranes developed when compared to fibers obtained with larger needle diameters [64].

- Collector

The type of collector utilized in the electrospinning process is a critical factor. The collector serves as a conductive substrate for the deposition of fibers in this method. Typically, the collector is covered with aluminum foil, although some authors have used alternative materials including conductive paper, wire mesh, or metal [65,66]. The collector can differ in shape, being a static collector, in the form of a flat metal plate, or a dynamic collector, such as a rotating metal cylinder, depending on the type of fiber to be obtained. The use of a flat plate makes it possible to obtain thicker membranes (using the same deposition time), but it has a small collection area and does not allow the fibers to be aligned. On the other hand, using the rotating cylinder makes it possible to obtain aligned fibers and, due to its larger collection area, to obtain membranes with larger fibers. However, there is some difficulty in guaranteeing the alignment of all of the fibers deposited, as well as the possibility of the fibers breaking if the rotation is too high [49]. In fact, several parameters influence the orientation of the fibers, such as the design and rotation of the collector. Several studies have evaluated these parameters and found that higher rotations are associated with greater fiber orientation [67,68].

#### 3.1.3. Environmental Parameters

The influence of environmental parameters is generally underestimated by many researchers, as only a few studies have monitored their effect on the electrospinning process. However, the structure and morphology of fibers are also affected by environmental conditions, namely temperature and humidity [69].

- Temperature

Temperature has a greater influence on the viscosity of the solution, since an increase in temperature leads to a decrease in the viscosity value and, consequently, a reduction in the average diameter of the nanofibers obtained. On the other hand, low temperatures tend to increase the viscosity of the solution, promoting the formation of fibers with larger diameters [69]. 

**Figure 8:**
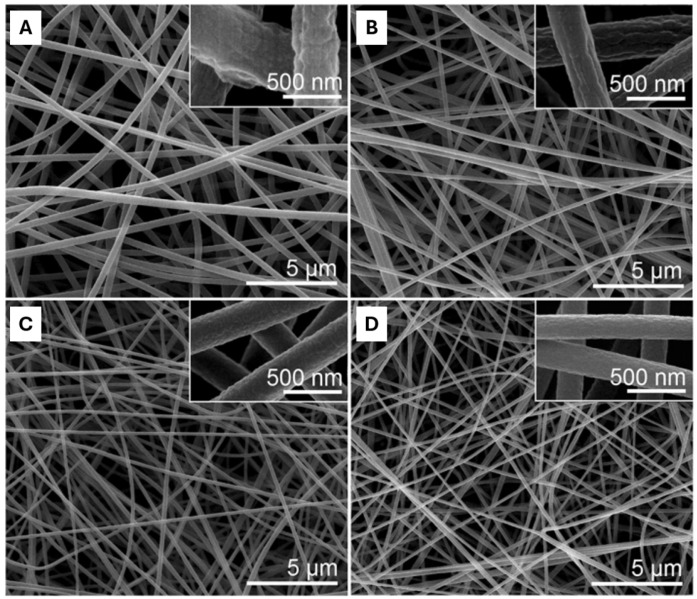
FESEM images of PAN/PVP nanofibers obtained at different ambient temperatures: (**A**) 20 °C; (**B**) 40 °C; (**C**) 60 °C; (**D**) 80 °C. Reprinted with permission from reference [71].

In a study by Mituppatham et al., the synthesis of polyamide fibers with thinner diameters was favored by an increase in temperature that was accompanied by a decrease in viscosity. The resulting nanofibers had a diameter of 98 nm at 30 °C and 90 nm at 60 °C, respectively [70]. Similar results were obtained by Yang et al. [71]. These authors studied the effect of temperature on polyacrylonitrile (PAN) and polyvinylpyrrolidone (PVP) fibers (Figure 8) and showed that in both cases, there was a gradual decrease in the diameter of the nanofibers from 530 to 280 nm for PAN fibers and from 830 to 540 nm for PVP fibers as the temperature increased from 20 °C to 60 °C and from 20 °C to 50 °C, respectively. However, for excessively high temperatures, it was found that the solvent evaporated too quickly and, consequently, the diameter of the fibers reached higher values.

In general, an increase in temperature favors the formation of fiber membranes with smaller average diameters. However, excessively high temperature values show the opposite effect, increasing the average diameter of the fibers [29].

- Humidity

Humidity is an environmental parameter that influences the final morphology of fibers produced by electrospinning. At low humidity values, the fibers dry out quickly due to an increase in the evaporation rate of the solvent. In addition, there is a chance that the fluid will dry up at the needle tip, which could cause issues with the electrospinning procedure. On the other hand, high humidity can promote the development of pores in the nanofibers and lead to an increase in their diameters [29,69]. 

**Figure 9:**
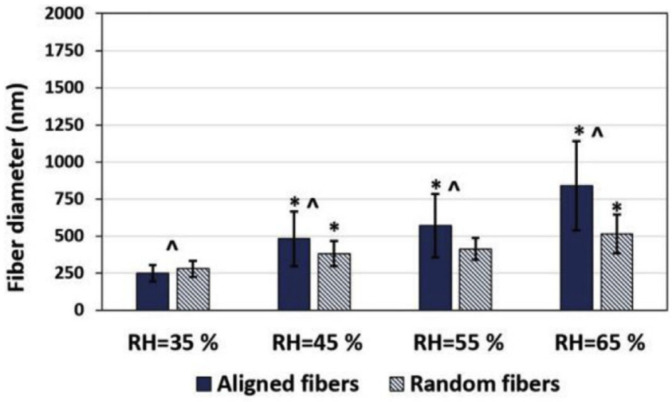
Effect of ambient humidity on the diameter of PCL fibers produced by electrospinning. (*: statistically significant difference between fibers electrospun with 2 different and consecutive parameters within the same aligned or random fiber group; ˆ: statistically significant difference between aligned and random fibers electrospun with the same parameter; *p* < 0.05). Reproduced with permission from reference [68].

Ghobeira et al. evaluated the effect of relative humidity on the diameter of PCL fibers, where they varied the humidity between 35% and 65%, keeping the other parameters constant. For a humidity value of 35%, the fibers had average diameters of 249 nm. As the humidity increased to 65%, there was a progressive increase in the diameter of the PCL fibers to 841 nm (Figure 9). This result can be explained by the presence of more water molecules between the needle and the collector, which reduces the load on the polymer jet. Consequently, there is a decrease in the intensity of the electric field, which will limit the proper elongation of the jet, resulting in fibers with larger diameters [68]. The effect of the humidity parameter was also evaluated by Casper et al. [72] and by Medeiros et al. for polystyrene fibers [73], where they found that increasing humidity promoted the production of nanofibers with larger diameters.

Thus, by examining previous studies, it can be concluded that in the electrospinning process, an increase in humidity favors the development of fibers with larger diameters, associated with an increase in water molecules in the atmosphere, and consequently results in changes in the elongation of the polymer jet. On the other hand, very low humidity values mean that the solvent evaporates very quickly, preventing the correct formation of the jet by drying the solution on the needle tip [29,74].

## 4. Electrospun Membranes for Water Treatment

The choice of polymers used in the electrospinning solution influences the solution’s viscosity, electrical conductivity, and surface tension, among other properties [75]. The surface tension of the spinning solution must be overcome by the supplied voltage, and lowering the surface tension promotes the creation of fibers free of granules [76].

Differentiated nanofibers and membranes are required for different applications, and the tunability of these materials may be limited by the use of a single polymer. By using the technology of electrospinning, polymers with various characteristics can be combined and spun together in a container to enhance membrane performance [77]. Because functionality from individual components can be integrated, nanofibers generated from a mixture of polymers can also lead to novel applications [76].

### 4.1. Synthetic Polymers

Among the various materials used to manufacture nanofibrous membranes by electrospinning, synthetic polymers including polyacrylonitrile (PAN), polyvinyl acetate (PVA), polyvinylpyrrolidone (PVP), polyetherimide (PEI), or polycaprolactone (PCL) have been the focus of much attention due to their physicochemical stability, film-forming properties, and spinning capacity. Table 1 shows some examples of the most common materials used to produce nanofibrous membranes with possible application in textile wastewater purification.

**Figure 10:**
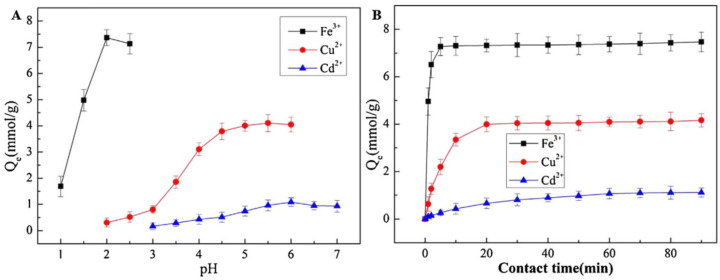
Effect of solution pH value (**A**) and contact time (**B**) on adsorption capacity of different heavy metal ions. During the experiments, the initial concentrations of Fe(III), Cu(II), and Cd(II) ions were 100 mmol/L. Each of the reported data is the average value of 3 replicas, and an error bar represents one standard deviation. Reprinted with permission from reference [87].

The electrospinning approach has effectively converted many synthetic polymers into nanofibers [103], with polyacrylonitrile (PAN) being of particular interest for the adsorption of heavy metal ions. This is due to the fact that amidoxime groups (-C(NH_2_)=NOH) are easily formed from nitrile groups (-C≡N) in PAN by reacting with hydroxylamine in an aqueous solution at room temperature [104,105,106]. The resulting amidoxime groups have a strong ability to adsorb heavy metal ions through the formation of coordination/chelation bonds [107,108,109]. PAN is usually combined with other materials to enhance its characteristics. In the study by Feng et al., an innovative nanomaterial was prepared and used for the removal of heavy metal ions from wastewater. Polyacrylonitrile/cellulose acetate (PAN/CA) nanofibrous membranes were created by electrospinning, and subsequently, amidoxime polyacrylonitrile/regenerated cellulose (AOPAN/RC) membranes were synthesized through a combination of hydrolysis and amidoximation modification. After characterizing the membranes, they found that the optimum solution pH values for the adsorption of Fe(III), Cu(II), and Cd(II) ions were 2, 5, and 6, respectively. At 25 °C, the nanofibrous membranes’ saturation adsorption amounts for Fe(III), Cu(II), and Cd(II) were 7.47, 4.26, and 1.13 mmol/g, respectively, with adsorption equilibrium being reached in 5, 20, and 60 min (Figure 10). After five consecutive adsorption and desorption tests, the desorption rate of the metal ions remained more than 80% of their first desorption rate [87]. 

Given its exceptional mechanical, thermal, and chemical resistance, polyacrylonitrile (PAN) has become a material that is frequently utilized in the water treatment industry. Since electrospun PAN nanofibers range in size from tens to hundreds of nanometers, they have excellent ion adsorption capability as well as tremendous potential for use in the adsorption of organic dyes due to their high surface area and better surface-to-volume ratio [110]. In a study by Du et al., RGO/TiO_2_/PANCMA composite nanofibers were obtained by electrospinning the dispersive solution of PANCMA, GO, and TiO_2_, followed by post-chemical reduction. Under optimized conditions, by E-spun RGO/TiO_2_/PANCMA nanofibers, 90.6% of malachite green and 93.7% of leucomalachite green were adsorbed in 2 min, and subsequently, 91.4% and 95.2% of adsorbed malachite green and leucomalachite green were degraded in 60 min under UV irradiation, respectively. In addition, the nanofibers showed good recyclability and can be reused in several cycles of operations for adsorption and photocatalytic degradation. In fact, before the 14th cycle, the removal efficiencies of malachite green and leucomalachite were over 91% [85]. Zhang et al. used the cationic dye methylene blue (MB) and the anionic dye methyl orange (MO) as model pollutants to investigate the photocatalytic activity of PAN/β-cyclodextrin (β-CD)/TiO_2_/GO nanofibrous membranes, which were produced by electrospinning in conjunction with ultrasound-assisted electrospinning. The membrane that demonstrated the highest dye degradation efficiency was the one with a minimum diameter of 84.66 ± 40.58 nm and an 8:2 mass ratio of TiO_2_ to GO. In 5 h, the degradation efficiencies for methyl orange and methylene blue were approximately 90.92% and 93.52%, respectively. The membranes maintained an 80% degradation efficiency of MB and MO over the course of three cycles, declining to around 68.42% and 65.13%, respectively, in the fifth cycle. Additionally, the PAN/β-CD/TiO_2_/GO membrane demonstrated antibacterial properties against *Staphylococcus aureus* and *Escherichia coli* [83].

The potential of metal–organic frameworks (MOFs) for dye adsorption is also very significant. However, the actual use of MOFs is hindered by the difficulty of removing MOF powder from an aqueous solution. The incorporation of MOFs into polymeric fiber membranes offers a novel approach to attain outcomes that preserve MOFs’ dye adsorption capability while facilitating the facile extraction of the MOF/dye complex from aqueous solutions. Ji et al. recently used the electrospinning method to successfully construct a zeolitic fibrous membrane of imidazole-67/polyacrylonitrile (ZIF-67/PAN) with a ZIF-67 loading ratio of 54%. The ZIF-67 and ZIF-67/PAN membranes have maximal malachite green adsorption capacities of 2545 and 1305 mg/g, respectively. The ZIF-67/PAN membrane retained more than 92% of its original fibers after four regeneration cycles. In addition, the ZIF-67/PAN fibers also showed good adsorption abilities for Congo red (849 mg/g) and fuchsin (730 mg/g). After being cleaned with ethanol, the membrane could be utilized again. Therefore, because of their straightforward manufacturing process, superior adsorption qualities, ease of separation, and advantageous reusability, ZIF-67/PAN fibers seem to be attractive adsorbents for the removal of dyes in industrial applications [82].

**Figure 11:**
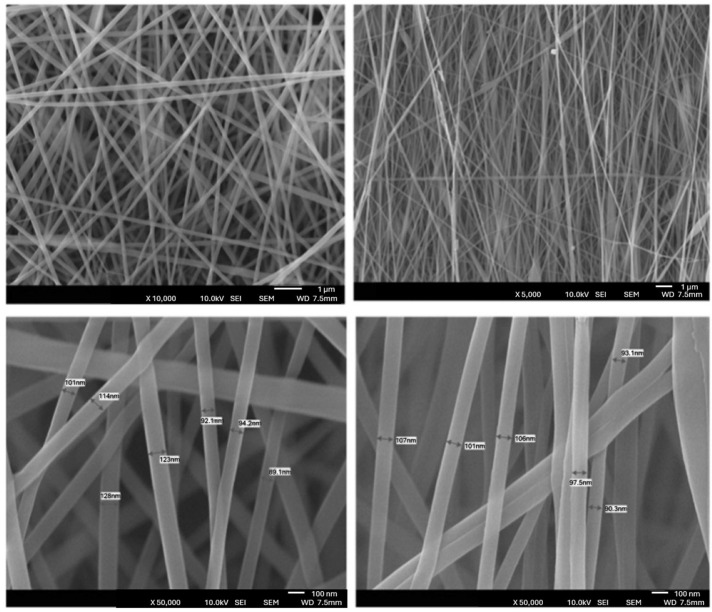
FESEM images of PVA/Chi nanofibers membranes. Adapted with permission from reference [93].

Polyvinyl acetate (PVA), a biocompatible polymer with good mechanical properties and biodegradability, has been widely used to form homogeneous miscible systems with strong chemical stability, film-forming ability, and high hydrophilicity. Furthermore, PVA’s side chain contains a greater number of hydroxyl groups, which increases its solubility in water [111]. Therefore, various materials can be hybridized with PVA to form nanofiber composites to be used as an economical adsorbent for ions and dyes from wastewater. As stated in an article by Karim et al., lead (Pb(II)) and cadmium (Cd(II)) ions were selectively and highly adsorbed onto nanofiber membranes made of polyvinyl acetate/chitosan (PVA/Chi) using the electrospinning technique, depending on how acidic the solution was (Figure 11).

**Figure 12:**
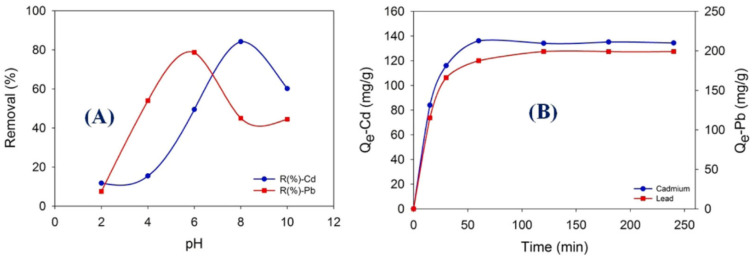
(**A**) Percentage adsorption of Cd(II) and Pb(II) ions at different pH ranges. (Conditions: 25 mg adsorbent, 10 mL of 400 mg/L solution of Pb(II)/Cd(II) ions, and contact time = 60 min.) (**B**) Adsorption of Cd(II) and Pb(II) ions on PVA/Chi NFs membrane. (Conditions: 0.025 g adsorbent, 10 mL of 400 mg/L solution of heavy-metal ions, and contact time = 5–240 min.) Reproduced with permission from reference [93].

Under ideal conditions, as seen in Figure 12, the maximum adsorption capacity was 266.12 mg/g (Pb(II)) and 148.79 mg/g (Cd(II)). For Cd(II) and Pb(II), in-depth adsorption investigations were conducted at pH 8 and 6, respectively. The PVA/Chi membranes’ ability to adsorb Pb(II) or Cd(II) ions was unaffected by foreign ions. Thus, PVA/Chi nanofiber membranes (70:30 PVA/Chi ratio) produced by electrospinning are considered effective and promising for removing Pb(II) and Cd(II) ions from wastewater with high efficiency. Furthermore, this method is a simpler and more sustainable process than conventional methods [93]. 

**Figure 13:**
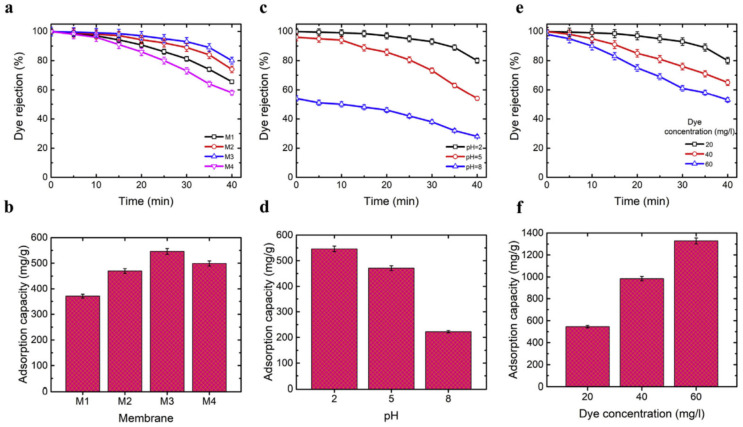
Effect of SiO_2_ concentration on (**a**) dye removal and (**b**) adsorption capacity (pH = 2 and dye concentration = 20 mg/L). Effect of pH on (**c**) dye removal and (**d**) adsorption capacity (dye concentration = 20 mg/L). Effect of dye concentration on (**e**) dye removal and (**f**) adsorption capacity (pH = 2). Reprinted with permission from reference [91].

Hosseini et al. developed innovative electrospun nanofibrous membranes with PVA, chitosan, and SiO_2_ nanocomposite materials to enhance their mechanical strength and permeability capabilities. The investigation focused on the morphology, fiber diameter, porosity, thermomechanical characteristics, and permeability of the synthesized membranes in relation to different concentrations of SiO_2_ in the spinning solution (0—M1, 0.5—M2, 1.0—M3, and 2.0—M4 wt%). The affinity membranes that were produced were used to extract dye from wastewater. It was discovered that adding SiO_2_ as a reinforcing ingredient increased the nanocomposite membranes’ resilience. The produced membranes’ Young’s modulus nearly doubled, from 0.74 MPa for PVA/chitosan to 1.69 MPa for nanocomposite membranes with the addition of 0.5% wt SiO_2_. The main discovery was that the addition of 1.0% wt SiO_2_ resulted in a significant improvement in dye rejection and water permeability, as seen in Figure 13. Under 0.4 bar transmembrane pressure, the improved nanocomposite membrane yielded 98% Direct Red 23 rejection with a water flux value as high as 1711 L/m^2^h. It was also discovered that the membranes were reusable and antifouling [91].

PVA/SiO_2_ composite nanofibers including cyclodextrin-functionalized mesostructures were synthesized, as reported by Teng et al. The preparation procedure combined the use of the electrospinning technique with the sol–gel process. The indigo carmine dye was effectively adsorbed by the PVA/SiO_2_ nanofiber membranes. In less than 40 min, the adsorption equilibrium was reached, and the maximum adsorption capacity was 495 mg/g. Furthermore, for practical usage, the membranes offer good recycling capabilities. It is also convenient to fix and recover the mesoporous membranes. As a result, this novel substance may offer a new method for removing dye molecules [92]. Lv and colleagues synthesized zinc oxide nanoparticle-loaded nanofiber membranes using PVA and konjac glucomannan (KGM) by means of environmentally friendly thermal crosslinking and green electrospinning. ZnO@PVA/KGM membranes exhibited a filtration performance of over 99.99% for ultrafine particles (300 nm), surpassing that of commercial HEPA filters. After 120 min of solar light, methyl orange was effectively decolorized with a clearance efficiency of more than 98% at an initial concentration of 20 mg/L. The resultant fibrous membranes exhibited enhanced photocatalytic and antibacterial activity against Escherichia coli and Bacillus subtilis, in addition to their efficiency in air filtration [94].

**Figure 14:**
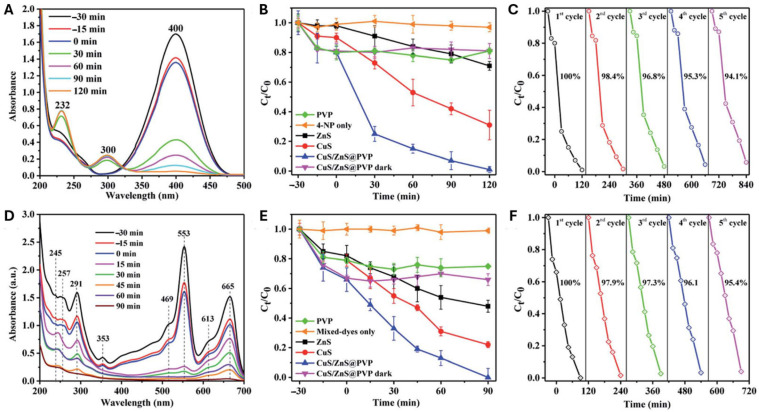
UV–vis spectra showing the conversion of (**A**) 4-nitrophenol and (**D**) mixed dyes. Plots of C_t_/C_0_ vs. reaction time comparing PVP only, organic pollutants only, and CuS, ZnS, and CuS/ZnS@PVP nanofibers to convert (**B**) 4-nitrophenol and (**E**) mixed dyes. The reusability performances after five cycles of CuS/ZnS@PVP nanofibers on (**C**) 4-nitrophenol and (**F**) mixed dyes. Reprinted with permission from reference [95].

Polyvinylpyrrolidone (PVP), a very water-soluble polymer, is used to produce systems that allow for the degradation of dyes from aqueous solutions, serving as a base for other components. Z-type-CuS/ZnS@PVP nanofibers were created by Sitinjak et al. using electrospinning with the intention of converting 4-nitrophenol to 4-aminophenol and degrading the mixtures of methyl orange, rhodamine B, and methylene blue when exposed to sun light. In 2 h, 4-nitrophenol was reduced to 4-aminophenol, and in 90 min, the combined dyes (10 ppm each) were completely degraded (Figure 14). The nanofibers showed excellent stability, with good reuse (94.1% after five cycles) of the catalyst [95].

In the work of Lu et al., zinc tungstate fibers (ZnWO_4_) with PVP were successfully produced by electrospinning. A total of 100 mL of aqueous rhodamine B solution (10 mg/L) was used to test the photocatalytic activity of the fibers under solar irradiation. The degradation efficiency was over 90% within about 90 min of irradiation. In addition, there was no decline in photocatalytic activity after five photodegradation cycles [98].

Not widely used in this area, polyarylene ether nitrile (PEN) is a type of high-performance thermoplastic with adaptable molecular structures, excellent mechanical properties, chemical and high-temperature resistance, good spinnability, and biocompatibility [112]. In the study by Li et al., nanofibrous membranes named PEN(B3S7), PEN(B5S5), and PEN(B7S3) were produced by electrospinning PEN, bisphenol (BPA), hydroquinone monosulfonic acid potassium salt (HQS), and 2,6-difluorobenzonitrile (DFBN). When it came to the cationic organic dye methylene blue, the optimized nanofibers demonstrated a high adsorption capacity of 796.25 mg/g. Even after eight separation–regeneration cycles, the cationic dyes’ removal efficiency reached 99% thanks to the improved polymer membrane, which enabled the quick and selective removal of the dyes from mixtures containing other dye molecules [102]. Thus, the optimized membranes made of PEN showed good performance in removing dyes under adverse conditions and an easy regeneration characteristic, as well as the possibility of reuse. Based on these characteristics, it is plausible to say that this observation provides new information for the development of advanced nanofibrous adsorbents and separation membranes for the purification of wastewater contaminated with organic dyes.

### 4.2. Natural Polymers

Bio-based polymers have been used as effective substitutes for synthetic polymers to reduce their negative effects on the environment because of their benign and ecological properties as well as their potential for commercial use [113]. With a unique chemical composition, biocompatibility, biodegradability, and functional chemical groups, such as hydroxyl, amine, and carboxyl groups, biopolymers have great potential for wastewater treatment by removing dyes and metal ions through various mechanisms, such as chelation, electrostatic attraction, and ion exchange [113,114].

#### 4.2.1. Cellulose Acetate

Cellulose acetate (CA) is the most researched derivative of cellulose since it may be deacetylated to regenerate pure cellulose. [115,116]. The process of acetylation, which involves substituting glucose units for cellulose hydroxyl groups to a degree of between two and four on average, produces cellulose acetate. For more than 70 years, CA has been effectively used in membrane filtration [117,118,119]. It has been extensively employed in nanofiltration and ultrafiltration as a selective layer. Considered among the most effective metal adsorbents, it has distinct functional groups that are modifiable [117,120]. The functionalization of CA with -COOH, -SO_3_H, and NH_2_ offers an opportunity for the application of CA in the complexation of heavy metals [121,122,123,124]. To improve a cellulose acetate membrane’s ability to adsorb metals, certain nanofillers can be added [117,121,122].

Taha et al. successfully produced cellulose acetate/silica nanofibrous membranes functionalized with NH_2_ through the sol–gel process combined with electrospinning technology. The membranes were used to remove Cr(VI) from aqueous solutions. The Langmuir adsorption model provides a good description of Cr(VI)’s adsorption behavior. At pH = 1, Cr(VI)’s maximum adsorption capacity was calculated to be 19.46 mg/g. After 60 min, for a Cr(VI) solution concentration of 10 ppm, the membrane’s adsorption efficiency was 98%. The membrane can also be regenerated through alkalinization [122]. In a project by Zayadi et al., a palm nanoleaf titanium fiber membrane (Nano-PFTF) was fabricated using cellulose acetate (CA) derived from palm leaf oil (OPF). The nanofiber membrane combined the adsorption and photocatalytic degradation of pollutants by nitrogen-doped titanium dioxide (N-TiO_2_). Under visible light and UV radiation, the Nano-PTFT membrane (CA/N-TiO_2_) achieved a 96.72% rejection of 10 ppm MB (methylene blue) and a 99% rejection of 10 ppm Cr(VI) in 120 min, respectively. In addition to separating the pollutants from the water, the membrane simultaneously reduced the pollutants with the presence of the photocatalyst N-TiO_2_ [125].

**Figure 15:**
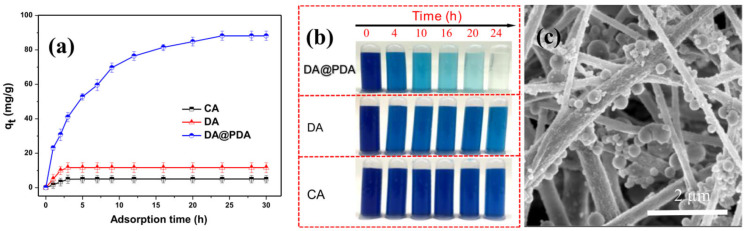
(**a**) Adsorption capacity of CA, DA, and DA@PDA nanofiber membranes with increasing adsorption time for the adsorption of MB dye. (**b**) Digital photographs of the MB solution after being immersed in the representative CA, DA, and DA@PDA nanofiber membranes. (**c**) SEM image of the DA@PDA composite nanofiber after MB adsorption for 24 h. (Adsorption conditions: original MB concentration was 50 mg/L, weight of adsorbent was 10 mg, temperature was 298 K, and pH was 6.5). Reproduced with permission from reference [126].

Cheng et al. successfully developed a uniform coating layer of polydopamine (PDA) on the surface of deacetylated cellulose acetate (DA) nanofibers using electrospinning. In order to extract methylene blue (MB) from an aqueous solution, the membrane was used as a highly effective adsorbent. After 30 h of adsorption at 25 °C and pH 6.5, the DA@PDA nanofiber membrane’s capacity to adsorb the MB dye was 88.2 mg/g, around 8.6 times higher than that of DA nanofibers, as seen in Figure 15 [126]. 

In a study by Akduman et al., cellulose acetate nanofiber membranes incorporated with diatomite (DE) were produced by electrospinning. A range of concentrations of 10, 20, and 30% DE (*w*/*w* polymer) was employed to investigate how different quantities of DE affected the removal of the dye Reactive Red 141. As a control, pure AC nanofibers were prepared by electrospinning. The equilibrium adsorption capacity of the AC, AC-10DE, AC-20DE, and AC-30DE nanofiber membranes after 24 h was found to be 66.26 ± 3.57, 67.83 ± 3.62, 70.71 ± 3.13, and 72.27 ± 2.90 mg/g, respectively, for an initial dye concentration of 85 mg/L [127].

Furthermore, San and colleagues discovered that the electrospun web of cellulose acetate nanofibers (AC-WNF) effectively immobilized bacterial cells. Three different species of bacteria (*Aeromonas eucrenophila*, *Clavibacter michiganensis*, and *Pseudomonas aeruginosa*) immobilized on AC-WNF were used to decolorize methylene blue (MB) dye in aqueous conditions. Effective decolorization of the MB dye was achieved in 24 h and the removal rate was 95%. The web showed good reusability; approximately 45% of the dye’s decolorization capacity was obtained at the end of the fourth cycle. More precisely, for *A. eucrenophila*, *C. michiganensis*, and *P. aeruginosa*, MB decolorization decreased to 45.7%, 43.1%, and 48.04%, respectively [128].

#### 4.2.2. Chitosan

Following cellulose, chitin is the second most prevalent natural polymer on the planet. Chitin is either deacetylated in an alkaline environment or hydrolyzed enzymatically in the presence of chitin deacetylase (CDA) to produce chitosan. It is an aminopolysaccharide with particular characteristics and functions that have a variety of uses, including industrial and biomedical. Chitosan is a copolymer composed of 2-acetamido-2-deoxy-β-D-glucopyranose and 2-amino-2-deoxy-β-glucopyranose connected by (1-4)-β-glycosidic linkages. For a variety of uses, many forms of chitin and chitosan have been created, including gels, membranes, spheres, microparticles, nanoparticles, and nanofibers [129,130]. Chitosan is well known for its unique antimicrobial activity and its capacity to adsorb metals [131,132].

**Figure 16:**
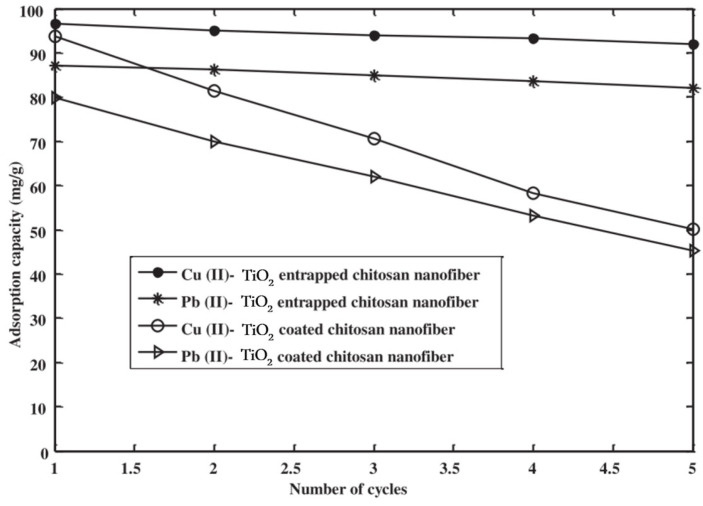
Five cycles of adsorption–desorption of Pb (II) and Cu (II) ions using chitosan/TiO_2_ nanofibrous adsorbents. Reprinted with permission from reference [135].

Li et al. produced pure chitosan membranes through electrospinning with average fiber diameters of 86 ± 18, 114 ± 17, and 164 ± 28 nm. Batch adsorption experiments were carried out using the chitosan nanofiber membranes as an adsorbent to remove acid blue-113. The chitosan membrane, with an average fiber diameter of 86 nm, had an adsorption capacity of 1377 mg/g, greater than that of the microscale chitosan sample, which had an adsorption capacity of 412 mg/g. Furthermore, following four cycles, the membranes demonstrated good regeneration. Adsorption capacity following the fourth regeneration cycle was 596.6 mg/g [133]. Likewise, Haider et al. produced chitosan membranes by electrospinning with nanofibers with a diameter of approximately 235 nm, but for the adsorption of ions from an aqueous solution. Cu(II) and Pb(II) had equilibrium adsorption capacities of 485.44 mg/g (2.85 mmol/g) and 263.15 mg/g (0.79 mmol/g), respectively, higher than the 45.20 mg/g value for the removal of Cu(II) ions from an aqueous solution using chitosan pellets [134]. Also, Razzaz et al. developed chitosan membranes with TiO_2_ nanoparticles (NPs) incorporated by electrospinning. In a batch setup, the produced nanofibers’ potential for Pb(II) and Cu(II) ion removal was examined. Cu(II) and Pb(II) ions had maximal adsorption capacities of 710.3 and 579.1 mg/g at 45 °C and 30 min, respectively, at equilibrium. The chitosan/TiO_2_ nanofibers could be reused frequently without significant loss of adsorption performance during five adsorption/desorption cycles (>80%), as shown in Figure 16 [135].

Mokhena et al. obtained a thin film of chitosan, with and without silver nanoparticles (AgNPs), on alginate nanofibers produced by electrospinning. Both membranes, with and without AgNPs, showed a similar flux and a high rejection of nanoparticles (>98%) and oil (>93%). Over the course of the five filtration cycles examined, the inclusion of AgNPs increased the dye Congo red’s rejection, achieving more than 95% rejection. The mem-branes demonstrated strong antibacterial action against Escherichia coli and Staphylococcus aureus as a result of the NPs [136].

#### 4.2.3. Alginate

Originating from seaweed, alginate is a widely recognized polyelectrolyte binary copolymer [137,138,139,140]. It contributes to the flexibility and resistance of marine algae against the adverse forces of water. It is typically found in the intercellular substance (mucilage) and cell wall matrix. Alginate is a linear polysaccharide consisting of 1–4 glycosidic linkages connecting D-mannuronic (M) and L-guluronic (G) units. These units depend on the source or species, growing conditions, season, and extraction depth, and they show up along the polymer chain in different sequences and proportions (M/G). The physical characteristics and reactivity of alginate are determined by the change in M and G along the alginate chain. It has been discovered that the affinity of alginates for heavy metals is significantly influenced by functional groups, particularly carbonate ions, and the molecule shape [140]. When alginate comes into contact with divalent or polyvalent metal ions, it can gel at ambient temperature. For specific applications, this phenomenon has been used to prepare various morphologies and structures. Spheres [141], films [142,143], and hydrogels [144], as well as porous membranes and nanofibers [138], have been manufactured for different applications, such as wound dressings and metal adsorption. Due to its antibacterial efficacy and structural resemblance to glycosaminoglycan (GAG), one of the important constituents of the natural extracellular matrix (EMC) present in mammalian tissues, alginate has attracted a great deal of interest for usage in biomedical applications. Furthermore, alginate’s availability, non-toxicity, biodegradability, and strong cell compatibility have made it possible to use it in a variety of applications, including metal recovery [140,141].

However, it remains controversial why it is not possible to carry out alginate electrospinning on its own. This has been attributed to the alginate solution’s strong conductivity, lack of entanglement, and gelation at low concentrations (below the concentration required for entanglement formation). This is further influenced by the strong surface tension, worm-like molecular structure, and stiffness of the chains. However, Mokhena et al. were able to produce alginate membranes by electrospinning for the biosorption of heavy metals from aqueous solutions. Since the electrospinnability of alginate from its aqueous solution is a problem, polyethylene glycol (PEO) was used to facilitate its spinnability. The membranes showed a maximum monolayer adsorption capacity (Q_0_) of 15.6 mg/g at a pH of 4 for Cu(II) ions. In a competitive experiment, the removal of metal ions in the mixture followed the order Cu > Ni > Cd > Co. The removal percentages were 39.2, 37.1, 25.3, and 21.8%, respectively. After five reuse cycles, the adsorption percentage had only decreased by 2% from the initial value [145].

Sodium alginate (SA) is a natural copolymer widely found in brown algae. This polymer’s outstanding biocompatibility, biodegradability, and non-toxicity make it useful in biological applications like tissue engineering, drug manufacturing, and other industrial uses. The production of adsorbents for use in adsorption operations is another usage for SA [146]. Unfortunately, because of its rigid structure, poor solubility, and high solution viscosity, sodium alginate presents several challenges when used as an adsorbent in the creation of nanofibers. Combining this polymer with others that have appropriate chain rotation and low viscosity, like polyvinyl acetate (PVA), is one way to solve this issue [147]. The study by Ebrahimi et al. aimed to manufacture nanofibers using polyvinyl acetate (PVA) and sodium alginate (SA) to remove cadmium metal ions from aqueous solutions. To this end, PVA/SA nanofibers (volume ratio 40:60) were produced by electrospinning. The maximum equilibrium adsorption amount for cadmium, under optimal experimental conditions, was 67.05 mg/g [148]. In addition, because sodium alginate is highly soluble in water, SA nanofiber membranes have inadequate stability in aqueous conditions. Thus, proper crosslinking is also a key factor in achieving the practical application of SA membranes in dye adsorption. Currently, the most used crosslinking technique for materials based on alginate is the use of calcium chloride (CaCl_2_). Wang and colleagues electrospun sodium alginate membranes, which were subsequently crosslinked using calcium chloride (CaCl_2_). The values of the SA electrospun nanofiber membranes’ surface area and pore volume before and after three different crosslinking techniques are displayed in Table 2.

**Figure 17:**
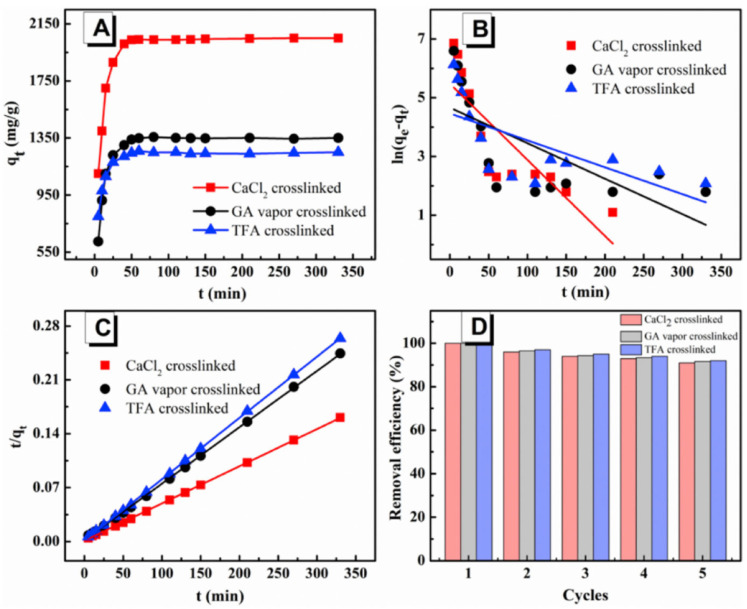
Adsorption kinetics (**A**) and the corresponding pseudo-first-order kinetic plots (**B**); pseudo-second-order kinetic plots (**C**); and adsorption–desorption cycles (**D**) for MB adsorption onto differentially crosslinked SA nanofiber membranes at 298 K. Reproduced with permission from reference [149].

All of the membranes showed excellent integrated adsorption performance for methylene blue (MB), with a maximum effective adsorption capacity of 2230 mg/g and an adsorption equilibrium time of 50 min (Figure 17). The methylene blue/methyl orange (MB/MO) mixture solution can be separated by the nanofiber membranes based on the selective adsorption of SA, and they can continue to separate the solution with a high separation efficiency even after five cycles (>90%) [149]. 

#### 4.2.4. β-Cyclodextrin

β-cyclodextrin (β-CD) is an important polysaccharide [150,151] that has been widely used as an adsorbent [152,153], since its macrocyclic structure can encapsulate a variety of hydrophobic molecules or parts of molecules inside the cavity through non-covalent interactions to form host–guest inclusion complexes [154,155]. It is not possible to directly remove target molecules from water using β-CD molecules since they are soluble in water. Therefore, grafting the β-CD molecules onto an existing substrate is required for water decontamination purposes [156,157] or to produce β-CD polymers that are insoluble [158,159,160]. 

**Figure 18:**
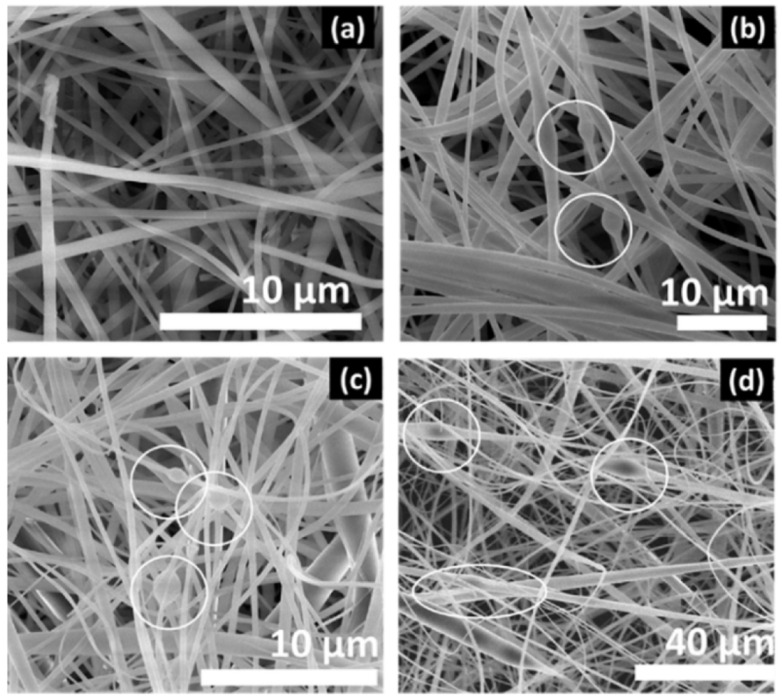
Representative SEM images of cyclodextrin fiber (CD-F) encapsulating (**a**) 0.25% (*w*/*w*), (**b**) 0.5% (*w*/*w*), (**c**) 1% (*w*/*w*), and (**d**) 2% (*w*/*w*) of bacteria. The circles show some of the encapsulated bacteria in the electrospun fiber matrix. Reprinted with permission from reference [161].

Cyclodextrin fibers produced by electrospinning (CD-F) are very attractive materials for encapsulating bacteria for bioremediation purposes. For instance, the encapsulated bacteria utilize cyclodextrin fibers as a food source, in addition to acting as a transport matrix. Keskin et al. demonstrated an easy approach to encapsulate bacteria in a cyclodextrin fiber matrix (CD-F), Figure 18, for application in wastewater treatment. *Lysinibacillus sphaericus*, the bacteria, were encased in cyclodextrin nanofibers made by means of electrospinning in order to treat textile dye (Reactive Black 5, RB5) and heavy metals (nickel (II) and chromium (VI)). The bacteria/CD biocomposite showed Ni(II), Cr(VI), and Reactive Black 5 dye removal efficiencies of 70 ± 0.2%, 58 ± 1.4%, and 82 ± 0.8%, respectively [161].

**Figure 19:**
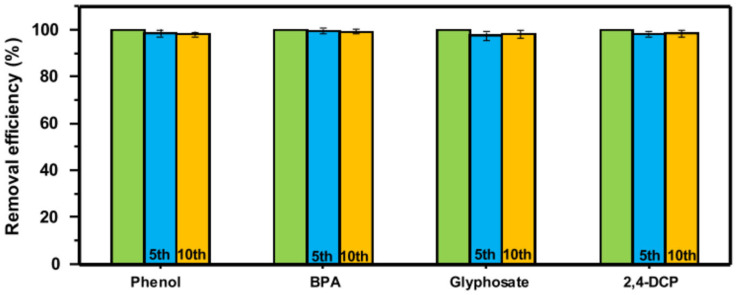
Removal efficiency of BC-CD-2 for each pollutant in the 5th and 10th cycle of the adsorption–desorption experiment. Reproduced with permission from reference [163].

Zhao et al. synthesized water-insoluble fibers based on β-cyclodextrin by electrospinning followed by thermal crosslinking. With good recyclability, the crosslinked fibers demonstrated a high adsorption capacity for the cationic dye methylene blue (MB). Considering the Langmuir model, the maximal adsorption capacity was 826.45 mg/g. The fibers exhibited poor adsorption toward the negatively charged anionic dye methyl orange (MO) as a result of electrostatic repulsion. The membrane could dynamically filter the MB/MO combination solution at a high flow rate of 150 mL/min based on selective adsorption. Even after five filtration–regeneration cycles (more than 90%), the fibers were still able to retain their excellent shape and great separation efficiency [162]. In their study, Liu et al. described a strategy for a film-type water purifier prepared by including a cyclodextrin oligomer (CD) in ultrathin bacterial cellulose (BC) nanofibers. The membrane showed a high adsorption capacity for four representative organic pollutants, including phenol, bisphenol A (BPA), glyphosate, and 2,4-dichlorophenol (2,4-DCP). The equilibrium adsorption capacity values were attained after 80 min for phenol, BPA, and 2,4-DCP, and 120 min for glyphosate. The values were 90.3 mg/g for phenol, 68.1 mg/g for BPA, 81.3 mg/g for glyphosate, and 222.6 mg/g for 2,4-DCP. The membrane maintained its superior adsorption ability in the presence of different anions and macromolecules and across a broad pH range. More importantly, it can be reused after treatment with methanol under ultrasonication. The removal efficiency in the 5th and 10th cycles (Figure 19) only showed a slight decrease (<5%) compared to the original removal efficiency [163]. When it comes to the number of target pollutants, including phenol, BPA, glyphosate, and 2,4-DCP, the ideal product exhibits an amazing removal capacity that surpasses the majority of adsorbents, including porous carbon-based materials, that have been previously documented.

## 5. Limitations of Textile Wastewater Filtering Structures by Electrospinning

The past decade has seen a significant increase in interest in advanced electrospinning technology because of its many benefits, including a wide range of material options, high porosity (>90%), and strong adaptability. Nevertheless, there are some limitations of this technique owing to the lengthy preparation cycle, the somewhat bigger average membrane pore size, the poor mechanical strength for modulation, making it difficult to produce a large mass volume of electrospun fibers, and long-term operation [6,164,165]. An electrospun nanofibrous membrane’s effectiveness might be impacted by several preparation and processing factors, as seen above. Polymer solution characteristics, operating circumstances, and environmental variables are the three primary categories. For instance, the characteristics and functionality of the membranes are greatly influenced by the pore size and thickness of the nanofibers. In general, when the fiber diameter is reduced, the membrane’s pore size shrinks as well, reducing water flux and increasing the salt interception rate [166,167,168]. Additionally, the high cost and accessibility of some of the used materials, the equipment (including maintenance), and the expert operators are among some major drawbacks of this technique [6,164]. 

Electrospun filtering structures may also show other limitations such as low pressure and temperature sensitivity, wettability, difficult cleaning, microorganism contaminations, and membrane fouling [169,170,171,172]. Membrane stability is also quite poorly addressed (there is a lack of long-term experiments in this regard) [164,173,174]. Another drawback of these materials is the use of non-environmentally friendly materials. However, this work already provides some insights into the use of eco-friendly natural polymers.

To increase the efficiency of electrospun membranes, the polymer materials and electrospinning procedures shall be further improved to overcome some of the mentioned limitations. The ecological impact of the membranes, such as their non-toxicity, long-term durability, and degradability properties, must be continuously optimized.

Besides the limitations of the developed structures, there are some literature gaps that shall be addressed. First, other types of polymers, both synthetic and natural, are quite unexplored for wastewater treatment applications. The lack of full-scale or even pilot-scale cases reveals a scalability issue that has to be addressed [25]. The small amount of pilot-scale reports lacks economic and environmental analysis [175,176,177]. Emerging technologies, such as electrodialysis and membrane bioreactors, are also under-addressed and should be considered in future works. 

## 6. Conclusions and Outlook

For a very long time, access to safe drinking water has been a serious issue across the world. Currently, one of the main sources of pollution is wastewater from textile dyeing. Various combinations of biological, physical, and chemical approaches are used in traditional technologies to treat textile effluent; however, these methods come with substantial capital and operating expenses. Membrane-based technologies provide one of the best alternatives available for large-scale environmentally sustainable treatment processes. In this regard, nanofibers have given the problem of water filtration a new dimension. These nanofibers offer advantageous qualities such as large surface areas, strength, and an ideal pore size. An easier and more practical way of creating nanofibers is electrospinning, which allows us to adjust the pore size of the nanofibers. In the last few decades, there have been innumerable advances in the field of electrospun nanofibrous membranes. Nanofibrous membranes for the treatment of water and wastewater are incredibly promising based on the present view of development and application. This technique offers access to a vast variety of nanomaterials with unique features, such as their eco-friendliness, degradability, renewability, non-toxicity, and mechanical and thermal properties, as well as their availability, long-term durability, and cost-effectiveness.

This paper summarizes the process variables that influence the electrospun nanofibers’ physical characteristics and functional capabilities, including large surface area, roughness, porosity, and surface chemistry (hydrophilicity/hydrophobicity), to offer ways to improve their efficacy in water and wastewater purification. We have discussed the most recent developments in the study of water purification using electrospun nanofibers. The most effective composite nanofibers for filtration applications include electrospun PAN, PVA, PVP, and CA nanofibers. The use of other natural polymers, such as chitosan or alginate, that can be extracted from natural sources is a growing field. Electrospun nanofibrous membranes based on these polymers have already been shown as powerful adsorbents for several metal ions and dyes, being a promising alternative to synthetic polymers for the development of textile wastewater filtering structures.

Despite their advantages, electrospun nanofibrous membranes are not without limitations. These include problems with inadequate nanoscale selectivity, mechanical weakness, high cost of and accessibility to the used materials, the equipment (including maintenance), and the expert operators. It is also important to note that an electrospun nanofibrous membrane’s effectiveness is impacted by several preparation and processing factors, such as room temperature and humidity. Another constraint is the lack of large-scale studies. Due to the variability in the composition of textile wastewater, the application of electrospun membrane systems in the processing of textile wastewater is difficult, which limits full-scale studies. The economic analysis of the scarce pilot-scale cases reported is insufficient, and the application of this type of material in real cases must be further studied. In order to develop affordable, industrial-scale modules and effective electrospun nanofibrous membranes for water treatment, research organizations and industrial businesses must work together to overcome many obstacles. Technical constraints during the fabrication and operation of nanofiber electrospun membranes, such as membrane stability and mechanical weakness, should be the subject of future research. Future works shall address the scalability issue, and joint efforts between scholars and the industry must be made. Economic and technical analyses are needed in pilot-scale reports, and the environmental impact of the methods and technologies developed should be considered. The use of natural fibers plays a crucial part in the development of more sustainable methodologies for water filtration. The recycling of not only textile residues but also natural (marine, agricultural, etc.) waste can contribute to the development of water treatment technologies in a circular way. 

Overall, the application of the electrospinning technique to water treatment technologies provides a great number of opportunities for effectively purifying textile wastewater, promising a cleaner and more sustainable future for the industry. Electrospun membranes do, in fact, have a bright future and are anticipated to play a significant role in the treatment of refractory contaminated water.

## Figures and Tables

**Table 1:** Examples of electrospun membranes for water treatment.

Membrane Composition	Results	Ref.
Polyacrylonitrile (PAN)	Retention of 99.99% of bacteriophages and 99.9999% of bacteria.	[78]
PAN Haloisite nanotubes (HNTs)	The incorporation of HNTs, especially 1% *w*/*w*, improved the mechanical and thermal properties of the membranes. Rejection rate of 99.5% of oil/water for membranes with 3% *w*/*w* HNTs, and removal efficiency of 31.1% of heavy metal ions.	[79]
PAN TIME Jute cellulose nanowhiskers	Good mechanical properties, efficient nanoparticle filtration capacity, and good oil/water separation (with a rejection rate of over 99%).	[80]
PAN Diethylenetriamine (DETA)	Maximum adsorption capacities: methylene blue—184.84 mg/g; rhodamine B—367.65 mg/g; safratin T—195.7 mg/g. The membrane showed higher maximum adsorption capacities when compared to conventional adsorbents.	[81]
PAN Structure of zeolitic imidazole-67 (ZIF-67)	Maximum adsorption capacities of malachite green: ZIF-67 membranes—2545 mg/g; ZIF-67/PAN membrane—1305 mg/g. After four regeneration cycles, the ZIF-67/PAN membrane showed more than 92% of its original capacity. It also showed good adsorption abilities for Congo red (849 mg/g) and fuchsin (730 mg/g). The membrane can be reused by washing it with ethanol.	[82]
PAN Graphene oxide (GO) Titanium dioxide (TiO_2_) β-cyclodextrin (β-CD)	In 5 h, the degradation efficiency for methyl orange and methylene blue was around 93.52% and 90.92%, respectively. The membranes’ MB and MO degradation efficiency was 80% for the first three cycles, but dropped to around 68.42% and 65.13% in the fifth cycle, respectively. Antibacterial properties against *E. coli* and *S. aureus*.	[83]
PAN Carbon nanotubes (CNTs)	Almost complete degradation after 120 min and 60 min for methylene blue and indigo carmine, respectively. Improvements of 38% and 84% in tensile strength and elastic modulus, respectively, with just 0.05 wt% CNTs.	[84]
Polyacrylonitrile-co-maleic acid (PANCMA) GO TiO_2_	Under optimized conditions, by the E-spun RGO/TiO_2_/PANCMA NFs, 90.6% of malachite green and 93.7% of leucomalachite green were adsorbed in 2 min, and subsequently 91.4% and 95.2% adsorbed were degraded in 60 min under UV irradiation, respectively. Good recyclability. Before the 14th cycle, the removal efficiencies of malachite green and leucomalachite were over 91%.	[85]
PAN GO	High rejection performance (almost 100% rejection of Congo red, 56.7% for Na_2_SO_4,_ and 9.8% for NaCl). The water flow under extremely low external pressure (1.0 bar) increased significantly due to the structure of the graphene oxide layer and the nanofibrous support.	[86]
PAN Cellulose acetate (CA)	The optimum solution pH values for the adsorption of Fe(III), Cu(II), and Cd(II) ions were 2, 5, and 6, respectively, and the adsorption equilibria were obtained in 5, 20, and 60 min. The amount of saturation adsorption of the nanofibrous membranes (at 25 °C) for Fe(III), Cu(II), and Cd(II) was 7.47, 4.26, and 1.13 mmol/g, respectively. After five consecutive adsorption and desorption tests, the desorption rate of the metal ions maintained more than 80% of their first desorption rate. The AOPAN/RC nanofibers showed excellent regeneration capacity.	[87]
PAN ZIF-8	With relatively larger surface areas (of 871.0 m^2^/g) and adequate pore sizes (from around 0.6 to 0.8 nm), the nanofibers exhibited greater Cr(VI) adsorption capacity (with q_max_ of 39.68 mg/g) and good recyclability.	[88]
PAN Polyaniline (PANI)	The maximum adsorption capacities for lead and Cr_2_O_7_^2−^ on the PANI-coated membranes were 290.12 and 1202.53 mg/g, respectively. Greater removal of lead ions (99%) compared to chromium (VI) ions (90%) at 5 mg/L. The PAN/PANI membrane retained almost 58% and 60% of its initial adsorption capacity after four cycles for Cr_2_O_7_^2−^ and Pb(II).	[89]
Polyvinyl acetate (PVA) 1,2,3,4 butanetetracarboxylic acid (BTAC) crosslinked	Good performance in adsorbing the dye Reactive red 141. The maximum adsorption capacity reached 88.31 mg/g. If the temperature is increased from 110 °C to 130 °C, the adsorption capacity decreases.	[90]
PVA Silica (SiO_2_) Chitosan	The addition of 1.0% wt SiO_2_ resulted in a significant improvement in dye rejection and water permeability. Under 0.4 bar transmembrane pressure, the improved nanocomposite membrane yielded 98% Direct Red 23 rejection with a water flux value as high as 1711 L/m^2^h. It was discovered that the membranes were reusable and antifouling.	[91]
PVA SiO_2_ Cyclodextrin	The maximum adsorption capacity for the indigo carmine dye reached 495 mg/g and adsorption equilibrium was reached in less than 40 min. Recycled through acidification.	[92]
PVA Chitosan	The maximum adsorption capacity was 266.12 mg/g (Pb(II)) and 148.79 mg/g (Cd(II)). Detailed adsorption studies were carried out at pH 8 and 6 for Cd(II) and Pb(II), respectively. It is a simpler and more sustainable process than conventional methods.	[93]
PVA Konjac glucomannan (KGM) Zinc oxide nanoparticles (ZnO NPs)	Filtration efficiency for ultrafine particles (300 nm) of over 99.99%, superior to commercial HEPA filters. Methyl orange removal efficiency of over 98%, with an initial concentration of 20 mg/L, during 120 min of solar irradiation. Antibacterial activity (*E. coli* and *Bacillus subtilis*).	[94]
Polyvinylpyrrolidone (PVP) Copper (II) acetate hydrate Zinc (II) acetate	A total of 100% degradation of mixed dyes (methylene blue, rhodamine B, and methyl orange, 10 ppm each) in 90 min. Good reusability (94.1% after five cycles).	[95]
PVP Graphitic carbon nitride (g-C_3_N_4_) Niobium pentoxide (Nb_2_O_5_)	After 120 min in visible light, 98.1% degradation was recorded for rhodamine B and phenol (10 mg/L each). No obvious change in the performance of the nanofibers was recorded after four cycles (remained ≈ 98%).	[96]
PVP Zinc oxide (ZnO) Bismuth oxide (Bi_2_O_3_)	The compound with a molar ratio of 23:1 (ZnO/Bi_2_O_3_) showed the best activity under both excitations (UV and visible light). Approximately 95% degradation of rhodamine B (1.0 × 10^−5^ M, 60 mL) was reported after 90 min.	[97]
PVP Zinc tungstate (ZnWO_4_)	The degradation efficiency of rhodamine B (10 mg/L) was over 90% in about 90 min of irradiation. There was no decline in photocatalytic activity after five photodegradation cycles.	[98]
Lacase Polyetherimide (PEI) Polycaprolactone (PCL)	After ten cycles, PCL/PEI/TTL and PCL/PEI/TVL had residual activities of 33.2 ± 0.2% and 26.0 ± 0.9%, respectively. At 50 °C and pH 5, PCL/PEI/TTL demonstrated the highest decolorization efficiency of orange II and malachite green, reaching over 86% and 46%, respectively, after eight continuous uses. PCL/PEI/TTL and PCL/PEI/TVL had maximum removal efficiencies of 64.5 ± 7.6% and 52.6 ± 0.1%, respectively, and successfully decomposed 2,6-dichlorophenol. Environmentally friendly, sustainable materials.	[99]
PEI TiO_2_	The PEI membrane modified with 0.2% TiO_2_ achieved a significant removal rate of *E. coli* (99%) and humic acid (≈80%). Degradation of 85% of methylene blue during the photocatalytic process.	[100]
Polystyrene (PS) GO	PSGO films had a removal capacity ≈ 2.3 times higher than that of pure PS membranes. After 120 min, the equilibrium value of the adsorption capacity (qe = 114 mg/g) was reached for all of the methylene blue concentrations that were examined. After the first cycle, the removal capacity was reduced to ≈65%, a value that became constant during subsequent cycles (up to a maximum of five cycles).	[101]
PEN Bisphenol A (BPA) Hydroquinone methanesulfonic acid potassium salt (HQS) 2,6-difluorobenzonitrile (DFBN)	Methylene blue exhibited a high adsorption capacity of 796.25 mg/g. Even after eight separation–regeneration cycles, the optimized membrane achieved a 99% selective removal efficiency of cationic dyes. Good recyclability and stability at high temperatures.	[102]

**Table 2:** BET analysis and mechanical property results of non-crosslinked and differentially crosslinked SA nanofiber membranes. Table reprinted with permission from reference [149].

Sample	Surface Area (m^2^/g)	Pore Volume (cm^3^/g)	Tensile Strength (MPa)	Elongation at Break (%)
Non-crosslinked	13.97	0.0256	3.8	9.8
CaCl_2_ crosslinked	13.56	0.0450	10.4	9.9
GA vapor crosslinked	11.86	0.0185	3.7	11.2
TFA crosslinked	15.26	0.0455	3.6	12.3

## Data Availability

No new data were created or analyzed in this study. Data sharing is not applicable to this article.

## References

[1] Sanaeepur H., Ebadi Amooghin A., Shirazi M.M.A., Pishnamazi M., Shirazian S. (2022). Water desalination and ion removal using mixed matrix electrospun nanofibrous membranes: A critical review. Desalination.

[2] Peydayesh M., Mezzenga R. (2021). Protein nanofibrils for next generation sustainable water purification. Nat. Commun..

[3] Adam M.R., Othman M.H.D., Kurniawan T.A., Puteh M.H., Ismail A.F., Khongnakorn W., Rahman M.A., Jaafar J. (2022). Advances in adsorptive membrane technology for water treatment and resource recovery applications: A critical review. J. Environ. Chem. Eng..

[4] Sultana M., Rownok M.H., Sabrin M., Rahaman M.H., Alam S.M.N. (2022). A review on experimental chemically modified activated carbon to enhance dye and heavy metals adsorption. Clean. Eng. Technol..

[5] Wang X., Hsiao B.S. (2016). Electrospun nanofiber membranes. Curr. Opin. Chem. Eng..

[6] Kugarajah V., Ojha A.K., Ranjan S., Dasgupta N., Ganesapillai M., Dharmalingam S., Elmoll A., Hosseini S.A., Muthulakshmi L., Vijayakumar S. (2021). Future applications of electrospun nanofibers in pressure driven water treatment: A brief review and research update. J. Environ. Chem. Eng..

[7] Wang Z., Xue M., Huang K., Liu Z. (2011). Textile Dyeing Wastewater Treatment. Advances in Treating Textile Effluent.

[8] Kant R. (2012). Textile dyeing industry an environmental hazard. Nat. Sci..

[9] Holkar C.R., Jadhav A.J., Pinjari D.V., Mahamuni N.M., Pandit A.B. (2016). A critical review on textile wastewater treatments: Possible approaches. J. Environ. Manag..

[10] Al-Ahmed Z.A., Al-Radadi N.S., Ahmed M.K., Shoueir K., El-Kemary M. (2020). Dye removal, antibacterial properties, and morphological behavior of hydroxyapatite doped with Pd ions. Arab. J. Chem..

[11] Siddique K., Rizwan M., Shahid M.J., Ali S., Ahmad R., Rizvi H. (2017). Textile Wastewater Treatment Options: A Critical Review. Enhancing Cleanup Environ. Pollut..

[12] Li Y., Zhu J., Cheng H., Li G., Cho H., Jiang M., Gao Q., Zhang X. (2021). Developments of Advanced Electrospinning Techniques: A Critical Review. Adv. Mater. Technol..

[13] Patel K.D., Kim H.W., Knowles J.C., Poma A. (2020). Molecularly Imprinted Polymers and Electrospinning: Manufacturing Convergence for Next-Level Applications. Adv. Funct. Mater..

[14] Nasreen S.A.A.N., Sundarrajan S., Nizar S.A.S., Ramakrishna S. (2019). Nanomaterials: Solutions to water-concomitant challenges. Membranes.

[15] Subrahmanya T.M., Arshad A.B., Lin P.T., Widakdo J., Makari H.K., Austria H.F.M., Hu C.C., Lai J.Y., Hung W.S. (2021). A review of recent progress in polymeric electrospun nanofiber membranes in addressing safe water global issues. RSC Adv..

[16] Zia Q., Tabassum M., Lu Z., Khawar M.T., Song J., Gong H., Meng J., Li Z., Li J. (2020). Porous poly(L–lactic acid)/chitosan nanofibres for copper ion adsorption. Carbohydr. Polym..

[17] Zhang K., Li Z., Deng N., Ju J., Li Y., Cheng B., Kang W., Yan J. (2019). Tree-like cellulose nanofiber membranes modified by citric acid for heavy metal ion (Cu^2+^) removal. Cellulose.

[18] Nalbandian M.J., Greenstein K.E., Shuai D., Zhang M., Choa Y.H., Parkin G.F., Myung N.V., Cwiertny D.M. (2015). Tailored synthesis of photoactive TiO_2_ nanofibers and Au/TiO_2_ nanofiber composites: Structure and reactivity optimization for water treatment applications. Environ. Sci. Technol..

[19] Thyavihalli Girijappa Y.G., Mavinkere Rangappa S., Parameswaranpillai J., Siengchin S. (2019). Natural Fibers as Sustainable and Renewable Resource for Development of Eco-Friendly Composites: A Comprehensive Review. Front. Mater..

[20] Yaseen D.A., Scholz M. (2019). Textile Dye Wastewater Characteristics and Constituents of Synthetic Effluents: A Critical Review.

[21] Wang X., Jiang J., Gao W. (2022). Reviewing textile wastewater produced by industries: Characteristics, environmental impacts, and treatment strategies. Water Sci. Technol..

[22] Malik A., Hussain M., Uddin F., Raza W., Hussain S., Habiba U.E., Malik T., Ajmal Z. (2021). Investigation of textile dyeing effluent using activated sludge system to assess the removal efficiency. Water Environ. Res..

[23] Dojčinović B.P., Obradović B.M., Kuraica M.M., Pergal M.V., Dolić S.D., Indić D.R., Tosti T.B., Manojlović D.D. (2016). Application of non-thermal plasma reactor for degradation and detoxification of high concentrations of dye Reactive Black 5 in water. J. Serbian Chem. Soc..

[24] Tijing L.D., Yao M., Ren J., Park C.H., Kim C.S., Shon H.K. (2019). Nanofibers for Water and Wastewater Treatment: Recent Advances and Developments.

[25] Ma Z., Chang H., Liang Y., Meng Y., Ren L., Liang H. (2024). Research progress and trends on state-of-the-art membrane technologies in textile wastewater treatment. Sep. Purif. Technol..

[26] Müller A.K., Xu Z.K., Greiner A. (2022). Filtration of Paint-Contaminated Water by Electrospun Membranes. Macromol. Mater. Eng..

[27] Nayl A.A., Abd-Elhamid A.I., Awwad N.S., Abdelgawad M.A., Wu J., Mo X., Gomha S.M., Aly A.A., Bräse S. (2022). Review of the Recent Advances in Electrospun Nanofibers Applications in Water Purification. Polymers.

[28] Mingjun C., Youchen Z., Haoyi L., Xiangnan L., Yumei D., Bubakir M.M., Weimin Y. (2019). An example of industrialization of melt electrospinning: Polymer melt differential electrospinning. Adv. Ind. Eng. Polym. Res..

[29] Haider A., Haider S., Kang I.K. (2018). A comprehensive review summarizing the effect of electrospinning parameters and potential applications of nanofibers in biomedical and biotechnology. Arab. J. Chem..

[30] Dadras Chomachayi M., Solouk A., Akbari S., Sadeghi D., Mirahmadi F., Mirzadeh H. (2018). Electrospun nanofibers comprising of silk fibroin/gelatin for drug delivery applications: Thyme essential oil and doxycycline monohydrate release study. J. Biomed. Mater. Res. Part A.

[31] Kalantari K., Afifi A.M., Jahangirian H., Webster T.J. (2019). Biomedical applications of chitosan electrospun nanofibers as a green polymer—Review. Carbohydr. Polym..

[32] Kyselica R., Enikov E.T., Polyvas P., Anton R. (2018). Electrostatic focusing of electrospun Polymer(PEO) nanofibers. J. Electrostat..

[33] Chen X., Xu C., He H. (2019). Electrospinning of silica nanoparticles-entrapped nanofibers for sustained gentamicin release. Biochem. Biophys. Res. Commun..

[34] Martins A., Reis R.L., Neves N.M. (2008). Electrospinning: Processing technique for tissue engineering scaffolding. Int. Mater. Rev..

[35] Zeyrek Ongun M., Paralı L., Oğuzlar S., Pechousek J. (2020). Characterization of β-PVDF-based nanogenerators along with Fe_2_O_3_ NPs for piezoelectric energy harvesting. J. Mater. Sci. Mater. Electron..

[36] Mirjalili M., Zohoori S. (2016). Review for application of electrospinning and electrospun nanofibers technology in textile industry. J. Nanostruct. Chem..

[37] Aruna S.T., Balaji L.S., Kumar S.S., Prakash B.S. (2017). Electrospinning in solid oxide fuel cells—A review. Renew. Sustain. Energy Rev..

[38] Pisani S., Dorati R., Conti B., Modena T., Bruni G., Genta I. (2018). Design of copolymer PLA-PCL electrospun matrix for biomedical applications. React. Funct. Polym..

[39] Costa R.G.F., De Oliveira J.E., De Paula G.F., De Picciani P.H.S., De Medeiros E.S., Ribeiro C., Mattoso L.H.C. (2012). Eletrofiação de polímeros em solução. Parte I: Fundamentação teórica. Polimeros.

[40] Leidy R., Maria Ximena Q.C. (2019). Use of electrospinning technique to produce nanofibres for food industries: A perspective from regulations to characterisations. Trends Food Sci. Technol..

[41] Mirtič J., Balažic H., Zupančič Š., Kristl J. (2019). Effect of Solution Composition Variables on Electrospun Alginate Nanofibers: Response Surface Analysis. Polymers.

[42] Dhandayuthapani B., Krishnan U.M., Sethuraman S. (2010). Fabrication and characterization of chitosan-gelatin blend nanofibers for skin tissue engineering. J. Biomed. Mater. Res. Part B Appl. Biomater..

[43] Nezarati R.M., Eifert M.B., Cosgriff-Hernandez E. (2013). Effects of Humidity and Solution Viscosity on Electrospun Fiber Morphology. Tissue Eng. Part C Methods.

[44] Li Z., Wang C. (2016). One-Dimensional Nanostructures Electrospinning Technique and Unique Nanofibers.

[45] Cheng Y.-L., Lee C.-Y., Huang Y.-L., Buckner C.A., Lafrenie R.M., Dénommée J.A., Caswell J.M., Want D.A., Gan G.G., Leong Y.C. (2016). Electrospinning and Drug Delivery.

[46] Sohi A.N., Naderi-Manesh H., Soleimani M., Mirzaei S., Delbari M., Dodel M. (2018). Influence of Chitosan Molecular Weight and Poly(ethylene oxide): Chitosan Proportion on Fabrication of Chitosan Based Electrospun Nanofibers. Polym. Sci. Ser. A.

[47] Colmenares-Roldán G.J., Quintero-Martínez Y., Agudelo-Gómez L.M., Rodríguez-Vinasco L.F., Hoyos-Palacio L.M. (2017). Influence of the molecular weight of polymer, solvents and operational condition in the electrospinning of polycaprolactone. Rev. Fac. Ing..

[48] Akduman Ç., Perrin E., Kumabasar A., Çay A. Effect of Molecular Weight on the Morphology of Electrospun Poly(Vinyl Alcohol) Nanofibers. Proceedings of the XIIIth International Izmir Textile and Apparel Symposium.

[49] Bhardwaj N., Kundu S.C. (2010). Electrospinning: A fascinating fiber fabrication technique. Biotechnol. Adv..

[50] Sill T.J., von Recum H.A. (2008). Electrospinning: Applications in drug delivery and tissue engineering. Biomaterials.

[51] Zuo W., Zhu M., Yang W., Yu H., Chen Y., Zhang Y. (2005). Experimental Study on Relationship Between Jet Instability and Formation of Beaded Fibers During Electrospinning. Polym. Eng. Sci..

[52] Mahmud M.M., Perveen A., Matin M.A., Arafat M.T. (2018). Effects of binary solvent mixtures on the electrospinning behavior of poly (vinyl alcohol). Mater. Res. Express.

[53] Gazquez G.C., Smulders V., Veldhuis S.A., Wieringa P., Moroni L., Boukamp B.A., Ten Elshof J.E. (2017). Influence of Solution Properties and Process Parameters on the Formation and Morphology of YSZ and NiO Ceramic Nanofibers by Electrospinning. Nanomaterials.

[54] Abid S., Hussain T. (2019). Materials Science & Engineering C Current applications of electrospun polymeric nano fi bers in cancer therapy. Mater. Sci. Eng. C.

[55] Haghju S., Bari M.R., Khaled-Abad M.A. (2018). Affecting parameters on fabrication of β-D-galactosidase immobilized chitosan/poly (vinyl alcohol) electrospun nanofibers. Carbohydr. Polym..

[56] Reda R.I., Wen M.M., El-Kamel A.H. (2017). Ketoprofen-loaded Eudragit electrospun nanofibers for the treatment of oral mucositis. Int. J. Nanomed..

[57] Megelski S., Stephens J.S., Bruce Chase D., Rabolt J.F. (2002). Micro- and nanostructured surface morphology on electrospun polymer fibers. Macromolecules.

[58] Fallah M., Bahrami S.H., Ranjbar-Mohammadi M. (2016). Fabrication and characterization of PCL/gelatin/curcumin nanofibers and their antibacterial properties. J. Ind. Text..

[59] Doshi J., Reneker D.H. (1995). Electrospinning process and applications of electrospun fibers. J. Electrostat..

[60] Patil J.V., Mali S.S., Kamble A.S., Hong C.K., Kim J.H., Patil P.S. (2017). Electrospinning: A versatile technique for making of 1D growth of nanostructured nanofibers and its applications: An experimental approach. Appl. Surf. Sci..

[61] Zargham S., Bazgir S., Tavakoli A., Rashidi A.S., Damerchely R. (2012). The Effect of Flow Rate on Morphology and Deposition Area of Electrospun Nylon 6 Nanofiber. J. Eng. Fiber. Fabr..

[62] Hekmati A.H., Rashidi A., Ghazisaeidi R., Drean J.Y. (2013). Effect of needle length, electrospinning distance, and solution concentration on morphological properties of polyamide-6 electrospun nanowebs. Text. Res. J..

[63] Kuchi C., Harish G.S., Reddy P.S. (2018). Effect of polymer concentration, needle diameter and annealing temperature on TiO_2_-PVP composite nanofibers synthesized by electrospinning technique. Ceram. Int..

[64] Abunahel B.M., Azman N.Z.N., Jamil M. (2018). Effect of Needle Diameter on the Morphological Structure of Electrospun n-Bi2O3/Epoxy-PVA Nanofiber Mats. Int. J. Chem. Mater. Eng..

[65] Wang X., Um I.C., Fang D., Okamoto A., Hsiao B.S., Chu B. (2005). Formation of water-resistant hyaluronic acid nanofibers by blowing-assisted electro-spinning and non-toxic post treatments. Polymer.

[66] Sundaray B., Subramanian V., Natarajan T.S., Xiang R.Z., Chang C.C., Fann W.S. (2004). Electrospinning of continuous aligned polymer fibers. Appl. Phys. Lett..

[67] Anindyajati A., Boughton P., Ruys A. (2015). The Effect of Rotating Collector Design on Tensile Properties and Morphology of Electrospun Polycaprolactone Fibres. MATEC Web Conf..

[68] Ghobeira R., Asadian M., Vercruysse C., Declercq H., De Geyter N., Morent R. (2018). Wide-ranging diameter scale of random and highly aligned PCL fibers electrospun using controlled working parameters. Polymer.

[69] De Vrieze S., Van Camp T., Nelvig A., Hagström B., Westbroek P., De Clerck K. (2009). The effect of temperature and humidity on electrospinning. J. Mater. Sci..

[70] Supaphol P., Mit-uppatham C., Nithitanakul M. (2005). Ultrafine Electrospun Polyamide-6 Fibers: Effects of Solvent System and Emitting Electrode Polarity on Morphology and Average Fiber Diameter. Macromol. Mater. Eng..

[71] Yang G.Z., Li H.P., Yang J.H., Wan J., Yu D.G. (2017). Influence of Working Temperature on The Formation of Electrospun Polymer Nanofibers. Nanoscale Res. Lett..

[72] Casper C.L., Stephens J.S., Tassi N.G., Chase D.B., Rabolt J.F. (2004). Controlling Surface Morphology of Electrospun Polystyrene Fibers: Effect of Humidity and MolecularWweight in the Electrospinning Process. Macromolecules.

[73] Medeiros E.S., Mattoso L.H.C., Offeman R.D., Wood D.F., Orts W.J. (2008). Effect of relative humidity on the morphology of electrospun polymer fibers. Can. J. Chem..

[74] Long Y.Z., Yan X., Wang X.X., Zhang J., Yu M. (2018). Electrospinning: The Setup and Procedure. Electrospinning: Nanofabrication and Applications.

[75] Herrero-Herreo M. (2021). Role of Electrospinning Parameters on Poly(Lactic-co-Glycolic Acid) and Poly(Caprolactone-co-Glycolic Acid) Membranes. Polymers.

[76] Tang Y., Cai Z., Sun X., Chong C., Yan X., Li M., Xu J. (2022). Electrospun Nanofiber-Based Membranes for Water Treatment. Polymers.

[77] Xue J., Wu T., Dai Y., Xia Y. (2019). Electrospinning and Electrospun Nanofibers: Methods, Materials, and Applications. Chem. Rev..

[78] Ma H., Hsiao B.S., Chu B. (2014). Functionalized electrospun nanofibrous microfiltration membranes for removal of bacteria and viruses. J. Membr. Sci..

[79] Makaremi M., De Silva R.T., Pasbakhsh P. (2015). Electrospun nanofibrous membranes of polyacrylonitrile/halloysite with superior water filtration ability. J. Phys. Chem. C.

[80] Cao X., Huang M., Ding B., Yu J., Sun G. (2013). Robust polyacrylonitrile nanofibrous membrane reinforced with jute cellulose nanowhiskers for water purification. Desalination.

[81] Haider S., Binagag F.F., Haider A., Mahmood A., Al Masry W.A., Alhoshan M., Khan S.U.D. (2015). Fabrication of the diethylenetriamine grafted polyacrylonitrile electrospun nanofibers membrane for the aqueous removal of cationic dyes. Sci. Adv. Mater..

[82] Jin L., Ye J., Wang Y., Qian X., Dong M. (2019). Electrospinning Synthesis of ZIF-67/PAN Fibrous Membrane with High-capacity Adsorption for Malachite Green. Fibers Polym..

[83] Zhang R., Ma Y., Lan W., Sameen D.E., Ahmed S., Dai J. (2021). Enhanced photocatalytic degradation of organic dyes by ultrasonic-assisted electrospray TiO_2_/graphene oxide on polyacrylonitrile/β-cyclodextrin nanofibrous membranes. Ultrason. Sonochem..

[84] Mohamed A., Yousef S., Ali Abdelnaby M., Osman T.A., Hamawandi B., Toprak M.S., Muhammed M., Uheida A. (2017). Photocatalytic degradation of organic dyes and enhanced mechanical properties of PAN/CNTs composite nanofibers. Sep. Purif. Technol..

[85] Du F., Sun L., Huang Z., Chen Z., Xu Z., Ruan G., Zhao C. (2020). Electrospun reduced graphene oxide/TiO_2_/poly (acrylonitrile-co-maleic acid) composite nanofibers for efficient adsorption and photocatalytic removal of malachite green and leucomalachite green. Chemosphere.

[86] Wang J., Zhang P., Liang B., Liu Y., Xu T., Wang L., Cao B., Pan K. (2016). Graphene Oxide as an Effective Barrier on a Porous Nanofibrous Membrane for Water Treatment. ACS Appl. Mater. Interfaces.

[87] Feng Q., Wu D., Zhao Y., Wei A., Wei Q., Fong H. (2018). Electrospun AOPAN/RC blend nanofiber membrane for efficient removal of heavy metal ions from water. J. Hazard. Mater..

[88] Yang X., Zhou Y., Sun Z., Yang C., Tang D. (2020). Effective strategy to fabricate ZIF-8@ZIF-8/polyacrylonitrile nanofibers with high loading efficiency and improved removing of Cr(VI). Colloids Surf. A Physicochem. Eng. Asp..

[89] Mohammad N., Atassi Y. (2021). Enhancement of removal efficiency of heavy metal ions by polyaniline deposition on electrospun polyacrylonitrile membranes. Water Sci. Eng..

[90] Akduman C., Akçakoca Kumbasar E.P., Morsunbul S. (2017). Electrospun nanofiber membranes for adsorption of dye molecules from textile wastewater. IOP Conf. Ser. Mater. Sci. Eng..

[91] Hosseini S.A., Vossoughi M., Mahmoodi N.M., Sadrzadeh M. (2018). Efficient dye removal from aqueous solution by high-performance electrospun nanofibrous membranes through incorporation of SiO_2_ nanoparticles. J. Clean. Prod..

[92] Teng M., Li F., Zhang B., Taha A.A. (2011). Electrospun cyclodextrin-functionalized mesoporous polyvinyl alcohol/SiO_2_ nanofiber membranes as a highly efficient adsorbent for indigo carmine dye. Colloids Surf. A Physicochem. Eng. Asp..

[93] Karim M.R., Aijaz M.O., Alharth N.H., Alharbi H.F., Al-Mubaddel F.S., Awual M.R. (2019). Composite nanofibers membranes of poly(vinyl alcohol)/chitosan for selective lead(II) and cadmium(II) ions removal from wastewater. Ecotoxicol. Environ. Saf..

[94] Lv D., Wang R., Tang G., Mou Z., Lei J., Han J., De Smedt S., Xiong R., Huang C. (2019). Ecofriendly Electrospun Membranes Loaded with Visible-Light-Responding Nanoparticles for Multifunctional Usages: Highly Efficient Air Filtration, Dye Scavenging, and Bactericidal Activity. ACS Appl. Mater. Interfaces.

[95] Sitinjak E.M., Masmur I., Marbun N.V.M.D., Hutajulu P.E., Gultom G., Sitanggang Y. (2022). Direct Z-scheme of n-type CuS/p-type ZnS@electrospun PVP nanofiber for the highly efficient catalytic reduction of 4-nitrophenol and mixed dyes. RSC Adv..

[96] Wang L., Li Y., Han P. (2021). Electrospinning preparation of g-C_3_N_4_/Nb_2_O_5_ nanofibers heterojunction for enhanced photocatalytic degradation of organic pollutants in water. Sci. Rep..

[97] Xing Y., Que W., Yin X., He Z., Liu X., Yang Y., Shao J., Kong L.B. (2014). Electrospun ZnO/Bi_2_O_3_ Nanofibers with Enhanced Photocatalytic Cctivity. Nanomaterials.

[98] Lu J., Liu M., Zhou S., Zhou X., Yang Y. (2017). Electrospinning fabrication of ZnWO4 nanofibers and photocatalytic performance for organic dyes. Dye. Pigment..

[99] Kolak S., Birhanlı E., Boran F., Bakar B., Ulu A., Yeşilada Ö., Ateş B. (2023). Tailor-made novel electrospun polycaprolactone/polyethyleneimine fiber membranes for laccase immobilization: An all-in-one material to biodegrade textile dyes and phenolic compounds. Chemosphere.

[100] Al-Ghafri B., Lau W.J., Al-Abri M., Goh P.S., Ismail A.F. (2019). Titanium dioxide-modified polyetherimide nanofiber membrane for water treatment. J. Water Process Eng..

[101] De Farias L.M.S., Ghislandi M.G., De Aguiar M.F., Silva B.R.S., Leal A.N.R., Silva F.D.A.O., Fraga T.J.M., De Melo C.P., Kleber G., Alves B. (2022). Electrospun polystyrene/graphene oxide fibers applied to the remediation of dye wastewater. Mater. Chem. Phys..

[102] Li X., Yi K., Ran Q., Fan Z., Liu C., Liu X., Jia K. (2022). Selective removal of cationic organic dyes via electrospun nanofibrous membranes derived from polyarylene ethers containing pendent nitriles and sulfonates. Sep. Purif. Technol..

[103] Greiner A., Wendorff J.H. (2007). Electrospinning: A fascinating method for the preparation of ultrathin fibers. Angew. Chem. Int. Ed..

[104] Lin W., Lu Y., Zeng H. (1993). Studies of the preparation, structure, and properties of an acrylic chelating fiber containing amidoxime groups. J. Appl. Polym. Sci..

[105] Zhang L., Luo J., Menkhaus T.J., Varadaraju H., Sun Y., Fong H. (2011). Antimicrobial nano-fibrous membranes developed from electrospun polyacrylonitrile nanofibers. J. Membr. Sci..

[106] Huang F., Xu Y., Liao S., Yang D., Hsieh Y.L., Wei Q. (2013). Preparation of amidoxime polyacrylonitrile chelating nanofibers and their application for adsorption of metal ions. Materials.

[107] Saeed K., Haider S., Oh T.J., Park S.Y. (2008). Preparation of amidoxime-modified polyacrylonitrile (PAN-oxime) nanofibers and their applications to metal ions adsorption. J. Membr. Sci..

[108] Wu Z., Zhang Y., Wang B., Qian G., Tao T. (2013). Synthesis of palladium dendritic nanostructures on amidoxime modified polyacrylonitrile fibers through a complexing-reducing method. Mater. Sci. Eng. B Solid-State Mater. Adv. Technol..

[109] Zhao H., Liu X., Yu M., Wang Z., Zhang B., Ma H., Wang M., Li J. (2015). A study on the degree of amidoximation of polyacrylonitrile fibers and its effect on their capacity to adsorb uranyl ions. Ind. Eng. Chem. Res..

[110] Jatoi A.W., Gianchandani P.K., Kim I.S., Ni Q.Q. (2019). Sonication induced effective approach for coloration of compact polyacrylonitrile (PAN) nanofibers. Ultrason. Sonochem..

[111] Hu X.Q., Ye D.Z., Tang J.B., Zhang L.J., Zhang X. (2016). From waste to functional additives: Thermal stabilization and toughening of PVA with lignin. RSC Adv..

[112] Lin G., Bai Z., Liu C., Liu S., Han M., Huang Y., Liu X. (2022). Mechanically robust, nonflammable and surface cross-linking composite membranes with high wettability for dendrite-proof and high-safety lithium-ion batteries. J. Membr. Sci..

[113] Phan D.N., Khan M.Q., Nguyen N.T., Phan T.T., Ullah A., Khatri M., Kien N.N., Kim I.S. (2021). A review on the fabrication of several carbohydrate polymers into nanofibrous structures using electrospinning for removal of metal ions and dyes. Carbohydr. Polym..

[114] Mokhena T.C., Jacobs V., Luyt A.S. (2015). A review on electrospun bio-based polymers for water treatment. Express Polym. Lett..

[115] Frey M.W. (2008). Electrospinning cellulose and cellulose derivatives. Polym. Rev..

[116] Sato A., Wang R., Ma H., Hsiao B.S., Chu B. (2011). Novel nanofibrous scaffolds for water filtration with bacteria and virus removal capability. J. Electron Microsc..

[117] Chu B., Brook S., Hsiao B.S., Ma H. (2009). High Flux High Efficiency Nanofiber Membranes and Methods of Production Thereof. No. 61/103,479. https://patentimages.storage.googleapis.com/e1/3a/7e/5f49ad5f2ac2dd/WO2010042647A2.pdf.

[118] Ma Z., Kotaki M., Ramakrishna S. (2005). Electrospun cellulose nanofiber as affinity membrane. J. Membr. Sci..

[119] Wenten I.G. (2003). Recent development in membrane science and its industrial applications. J. Sci. Technol..

[120] Ma H., Burger C., Hsiao B.S., Chu B. (2012). Highly permeable polymer membranes containing directed channels for water purification. ACS Macro Lett..

[121] Ji F., Li C., Tang B., Xu J., Lu G., Liu P. (2012). Preparation of cellulose acetate/zeolite composite fiber and its adsorption behavior for heavy metal ions in aqueous solution. Chem. Eng. J..

[122] Taha A.A., Wu Y., Wang H., Li F. (2012). Preparation and application of functionalized cellulose acetate/silica composite nanofibrous membrane via electrospinning for Cr(VI) ion removal from aqueous solution. J. Environ. Manag..

[123] Bódalo A., Gómez J.L., Gómez E., León G., Tejera M. (2005). Ammonium removal from aqueous solutions by reverse osmosis using cellulose acetate membranes. Desalination.

[124] Konwarh R., Karak N., Misra M. (2013). Electrospun cellulose acetate nanofibers: The present status and gamut of biotechnological applications. Biotechnol. Adv..

[125] Ismail I.I.N., Zayadi R.A., Ho K.C., Soo J.Z., Idris M.S., Tay K.Y. (2020). Electrospun Nano-Palm Frond Titania Fiber (Nano-PFTF) Membrane for Industrial Wastewater Treatment. Solid State Sci. Technol..

[126] Cheng J., Zhan C., Wu J., Cui Z., Si J., Wang Q., Peng X., Turng L.S. (2020). Highly Efficient Removal of Methylene Blue Dye from an Aqueous Solution Using Cellulose Acetate Nanofibrous Membranes Modified by Polydopamine. ACS Omega.

[127] Akduman Ç. (2019). Fabrication and characterization of diatomite functionalized cellulose acetate nanofibers. AATCC J. Res..

[128] San N.O., Celebioglu A., Tümtaş Y., Uyar T., Tekinay T. (2014). Reusable bacteria immobilized electrospun nanofibrous webs for decolorization of methylene blue dye in wastewater treatment. RSC Adv..

[129] Schiffman J.D., Schauer C.L. (2008). A review: Electrospinning of biopolymer nanofibers and their applications. Polym. Rev..

[130] Jayakumar R., Menon D., Manzoor K., Nair S.V., Tamura H. (2010). Biomedical applications of chitin and chitosan based nanomaterials—A short review. Carbohydr. Polym..

[131] Muzzarelli R.A.A. (2011). Potential of chitin/chitosan-bearing materials for uranium recovery: An interdisciplinary review. Carbohydr. Polym..

[132] Wan Ngah W.S., Teong L.C., Hanafiah M.A.K.M. (2011). Adsorption of dyes and heavy metal ions by chitosan composites: A review. Carbohydr. Polym..

[133] Li C., Lou T., Yan X., Long Y., Cui G., Wang X. (2018). Fabrication of pure chitosan nanofibrous membranes as effective absorbent for dye removal. Int. J. Biol. Macromol..

[134] Haider S., Park S.Y. (2009). Preparation of the electrospun chitosan nanofibers and their applications to the adsorption of Cu(II) and Pb(II) ions from an aqueous solution. J. Membr. Sci..

[135] Razzaz A., Ghorban S., Hosayni L., Irani M., Aliabadi M. (2016). Chitosan nanofibers functionalized by TiO_2_ nanoparticles for the removal of heavy metal ions. J. Taiwan Inst. Chem. Eng..

[136] Mokhena T.C., Luyt A.S. (2017). Development of multifunctional nano/ultrafiltration membrane based on a chitosan thin film on alginate electrospun nanofibres. J. Clean. Prod..

[137] Alsberg E., Anderson K.W., Albeiruti A., Franceschi R.T., Mooney D.J. (2001). Cell-interactive alginate hydrogels for bone tissue engineering. J. Dent. Res..

[138] Dar A., Shachar M., Leor J., Cohen S. (2002). Optimization of cardiac cell seeding and distribution in 3D porous alginate scaffolds. Biotechnol. Bioeng..

[139] Hashimoto T., Suzuki Y., Tanihara M., Kakimaru Y., Suzuki K. (2004). Development of alginate wound dressings linked with hybrid peptides derived from laminin and elastin. Biomaterials.

[140] Davis T.A., Volesky B., Mucci A. (2003). A review of the biochemistry of heavy metal biosorption by brown algae. Water Res..

[141] Papageorgiou S.K., Katsaros F.K., Kouvelos E.P., Kanellopoulos N.K. (2009). Prediction of binary adsorption isotherms of Cu2+, Cd2+ and Pb2+ on calcium alginate beads from single adsorption data. J. Hazard. Mater..

[142] Xiao C., Liu H., Lu Y., Zhang L. (2000). Preparation and Physical Properties of Blend Films from Sodium Alginate and Polyacrylamide Solutions. J. Macromol. Sci. Part A Pure Appl. Chem..

[143] Çaykara T., Demirci S., Eroǧlu M.S., Güven O. (2005). Poly(ethylene oxide) and its blends with sodium alginate. Polymer.

[144] Omidian H., Rocca J.G., Park K. (2006). Elastic, superporous hydrogel hybrids of polyacrylamide and sodium alginate. Macromol. Biosci..

[145] Mokhena T.C., Jacobs N.V., Luyt A.S. (2017). Electrospun alginate nanofibres as potential bio-sorption agent of heavy metals in water treatment. Express Polym. Lett..

[146] Fang D., Liu Y., Jiang S., Nie J., Ma G. (2011). Effect of intermolecular interaction on electrospinning of sodium alginate. Carbohydr. Polym..

[147] Li W., Li X., Chen Y., Li X., Deng H., Wang T., Huang R., Fan G. (2013). Poly(vinyl alcohol)/sodium alginate/layered silicate based nanofibrous mats for bacterial inhibition. Carbohydr. Polym..

[148] Ebrahimi F., Sadeghizadeh A., Neysan F., Heydari M. (2019). Fabrication of nanofibers using sodium alginate and Poly(Vinyl alcohol) for the removal of Cd2+ ions from aqueous solutions: Adsorption mechanism, kinetics and thermodynamics. Heliyon.

[149] Wang Q., Ju J., Tan Y., Hao L., Ma Y., Wu Y., Zhang H., Xia Y., Sui K. (2019). Controlled synthesis of sodium alginate electrospun nanofiber membranes for multi-occasion adsorption and separation of methylene blue. Carbohydr. Polym..

[150] Szejtli J. (1998). Introduction and general overview of cyclodextrin chemistry. Chem. Rev..

[151] Crini G. (2014). Review: A history of cyclodextrins. Chem. Rev..

[152] Liu H., Cai X., Wang Y., Chen J. (2011). Adsorption mechanism-based screening of cyclodextrin polymers for adsorption and separation of pesticides from water. Water Res..

[153] Liu J., Liu G., Liu W. (2014). Preparation of water-soluble β-cyclodextrin/poly(acrylic acid)/graphene oxide nanocomposites as new adsorbents to remove cationic dyes from aqueous solutions. Chem. Eng. J..

[154] Connors K.A. (1997). The stability of cyclodextrin complexes in solution. Chem. Rev..

[155] Sherje A.P., Dravyakar B.R., Kadam D., Jadhav M. (2017). Cyclodextrin-based nanosponges: A critical review. Carbohydr. Polym..

[156] Liu F., Sun Y., Gu J., Gao Q., Sun D., Zhang X., Pan B., Qian J. (2020). Highly efficient photodegradation of various organic pollutants in water: Rational structural design of photocatalyst via thiol-ene click reaction. Chem. Eng. J..

[157] Liu Q., Zhou Y., Lu J., Zhou Y. (2020). Novel cyclodextrin-based adsorbents for removing pollutants from wastewater: A critical review. Chemosphere.

[158] Alsbaiee A., Smith B.J., Xiao L., Ling Y., Helbling D.E., Dichtel W.R. (2016). Rapid removal of organic micropollutants from water by a porous β-cyclodextrin polymer. Nature.

[159] Morin-Crini N., Winterton P., Fourmentin S., Wilson L.D., Fenyvesi É., Crini G. (2018). Water-insoluble β-cyclodextrin–epichlorohydrin polymers for removal of pollutants from aqueous solutions by sorption processes using batch studies: A review of inclusion mechanisms. Prog. Polym. Sci..

[160] Jeong D., Joo S.W., Shinde V.V., Jung S. (2018). Triple-crosslinkedβ-cyclodextrin oligomer self-healing hydrogel showing high mechanical strength, enhanced stability and pH responsiveness. Carbohydr. Polym..

[161] San Keskin N.O., Celebioglu A., Sarioglu O.F., Uyar T., Tekinay T. (2018). Encapsulation of living bacteria in electrospun cyclodextrin ultrathin fibers for bioremediation of heavy metals and reactive dye from wastewater. Colloids Surf. B Biointerfaces.

[162] Zhao R., Wang Y., Li X., Sun B., Wang C. (2015). Synthesis of β-cyclodextrin-based electrospun nanofiber membranes for highly efficient adsorption and separation of methylene blue. ACS Appl. Mater. Interfaces.

[163] Liu F., Chen C., Qian J. (2021). Film-like bacterial cellulose/cyclodextrin oligomer composites with controllable structure for the removal of various persistent organic pollutants from water. J. Hazard. Mater..

[164] Chen H., Huang M., Liu Y., Meng L., Ma M. (2020). Functionalized electrospun nanofiber membranes for water treatment: A review. Sci. Total Environ..

[165] Brown T.D., Dalton P.D., Hutmacher D.W. (2016). Melt electrospinning today: An opportune time for an emerging polymer process. Prog. Polym. Sci..

[166] Jian S., Zhu J., Jiang S., Chen S., Fang H., Song Y., Duan G., Zhang Y., Hou H. (2018). Nanofibers with diameter below one nanometer from electrospinning. RSC Adv..

[167] Xue J., Xie J., Liu W., Xia Y. (2017). Electrospun Nanofibers: New Concepts, Materials, and Applications. Acc. Chem. Res..

[168] Kaur S., Sundarrajan S., Rana D., Matsuura T., Ramakrishna S. (2012). Influence of electrospun fiber size on the separation efficiency of thin film nanofiltration composite membrane. J. Membr. Sci..

[169] Dobosz K.M., Kuo-Leblanc C.A., Martin T.J., Schiffman J.D. (2017). Ultrafiltration Membranes Enhanced with Electrospun Nanofibers Exhibit Improved Flux and Fouling Resistance. Ind. Eng. Chem. Res..

[170] Park M.J., Gonzales R.R., Abdel-Wahab A., Phuntsho S., Shon H.K. (2018). Hydrophilic polyvinyl alcohol coating on hydrophobic electrospun nanofiber membrane for high performance thin film composite forward osmosis membrane. Desalination.

[171] Shokrollahzadeh S., Tajik S. (2018). Fabrication of thin film composite forward osmosis membrane using electrospun polysulfone/polyacrylonitrile blend nanofibers as porous substrate. Desalination.

[172] Zhou T., Li J., Guo X., Yao Y., Zhu P., Xiang R. (2019). Freestanding PTFE electrospun tubular membrane for reverse osmosis brine concentration by vacuum membrane distillation. Desalin. Water Treat..

[173] Xu Y., Yang Y., Fan X., Liu Z., Song Y., Wang Y., Tao P., Song C., Shao M. (2021). In-situ silica nanoparticle assembly technique to develop an omniphobic membrane for durable membrane distillation. Desalination.

[174] Gontarek-Castro E., Castro-Muñoz R., Lieder M. (2022). New insights of nanomaterials usage toward superhydrophobic membranes for water desalination via membrane distillation: A review. Crit. Rev. Environ. Sci. Technol..

[175] Ağtaş M., Yılmaz Ö., Dilaver M., Alp K., Koyuncu İ. (2021). Pilot-scale ceramic ultrafiltration/nanofiltration membrane system application for caustic recovery and reuse in textile sector. Environ. Sci. Pollut. Res..

[176] Sahinkaya E., Tuncman S., Koc I., Guner A.R., Ciftci S., Aygun A., Sengul S. (2019). Performance of a pilot-scale reverse osmosis process for water recovery from biologically-treated textile wastewater. J. Environ. Manag..

[177] Kurt E., Koseoglu-Imer D.Y., Dizge N., Chellam S., Koyuncu I. (2012). Pilot-scale evaluation of nanofiltration and reverse osmosis for process reuse of segregated textile dyewash wastewater. Desalination.

